# HRS Degradation‐Induced Nicotinamide Deficiency in Placental Extracellular Vesicles Triggers Preeclampsia by Disrupting Maternal‐Fetal Immune Homeostasis

**DOI:** 10.1002/advs.202510188

**Published:** 2026-01-14

**Authors:** Haiyi Fei, Yuhan Lin, Xiu Liu, Xiaohong Zhu, Xiaowen Lu, Zhan Shi, Fan Jia, Shiqian Xu, Ling Fang, Lingling Jiang, Songying Zhang

**Affiliations:** ^1^ Assisted Reproduction Unit Department of Obstetrics and Gynecology School of Medicine Sir Run Run Shaw Hospital Zhejiang University Hangzhou China; ^2^ Zhejiang Provincial Clinical Research Center For Reproductive Health and Disease Hangzhou China; ^3^ Zhejiang Key Laboratory of Precise Protection and Promotion of Fertility Hangzhou China; ^4^ Department of Obstetrics and Gynecology Zhejiang Xiaoshan Hospital Hangzhou China; ^5^ Department of Cardiology Sir Run Run Shaw Hospital Zhejiang University School of Medicine Hangzhou China

**Keywords:** extracellular vesicles, HRS, HSP27, immune tolerance, nicotinamide, preeclampsia

## Abstract

Preeclampsia (PE) is closely associated with alterations in placental extracellular vesicles (pEVs), but the mechanisms and their role in PE pathogenesis remain unclear. This study reveals that nicotinamide (NAM) levels in PE‐derived pEVs (PE‐EVs) are lower than in pEVs from normal pregnancies, correlating with disease severity. Functionally, NAM in pEVs inhibits Th1 differentiation via SIRT1 suppression and Th17 differentiation via macrophages. NAM‐deficient pEVs exhibit reduced capacity to inhibit Th1 and Th17 cell differentiation both in vitro and in vivo, leading to PE‐like symptoms. NAM is enriched in pEVs compared to placental villous tissue and maternal serum. The lower NAM in PE‐EVs is due to decreased hepatocyte growth factor‐regulated tyrosine kinase substrate (HRS) expression in trophoblasts, which loads NAM into the cargo of multivesicular bodies (MVBs) via binding to the tryptophan‐115 residue of HRS. Furthermore, the reduction of HRS in PE trophoblasts results from ubiquitination and degradation by elevated HSP27. Collectively, these findings indicate that elevated HSP27 in PE trophoblasts leads to the degradation of HRS, a reduction in pEV NAM levels, and diminished Th1 and Th17 inhibitory effects, thereby contributing to the development of PE.

## Graphical abstract

This study shows that lower NAM levels in PE‐derived pEVs correlate with disease severity. NAM‐deficient pEVs reduce Th1 and Th17 inhibition, leading to PE‐like symptoms. NAM in pEVs inhibits Th1 via SIRT1 and Th17 via macrophages. Reduced NAM in PE‐EVs is due to decreased HRS expression in trophoblasts, resulting from elevated HSP27. These changes contribute to PE development.

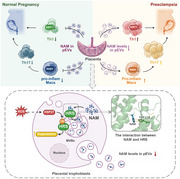

## 1 Introduction

Preeclampsia (PE), a pregnancy‐specific multisystem disorder affecting 3%–8% of global pregnancies, is characterized by new‐onset hypertension, multiorgan dysfunction, and placental insufficiency [1, 2]. PE is a leading cause of maternal and fetal mortality, with no effective treatment currently available. The only definitive solution is the delivery of the fetus and placenta, underscoring its nature as a placental disease [1]. However, the mechanisms by which the placenta triggers PE remain unclear.

Placental extracellular vesicles (pEVs)—primarily derived from trophoblasts and marked by placental alkaline phosphatase (PLAP)—play essential roles in intercellular communication [3, 4]. Disruption of placental immune homeostasis is a well‐established contributor to the pathogenesis of PE [5], and alterations in pEVs have recently emerged as a critical focus in understanding this condition [3, 6]. Comparative studies have revealed significant differences in the quantity, composition, and biological functions of pEVs from normal pregnancies (NP‐EVs) and those from PE pregnancies (PE‐EVs) [3]. For example, PE‐derived pEVs exhibit elevated levels of active Neprilysin—a metalloprotease implicated in hypertension, heart failure, and amyloid deposition—suggesting a potential mechanistic link to hypertensive manifestations of PE [7]. Additionally, the lipid profile of PE‐EVs is altered, with increased abundance of lipid species associated with oxidative stress, apoptosis, immune activation, and coagulation. These changes may promote abnormal macrophage polarization, though this hypothesis requires further validation [8]. Awoyemi et al. demonstrated that pEVs isolated from placental perfusate of PE patients suppress immune responses in THP‐1 macrophages, although the specific vesicular components responsible remain unidentified [9]. Furthermore, syncytiotrophoblast‐derived EVs are known to regulate extravillous trophoblast invasion function within the decidua [10]. Impaired trophoblasts invasion is a hallmark of defective placentation in PE [11], suggesting that aberrant EV‐mediated signaling may contribute to this dysfunction. In summary, despite growing evidence linking pEV cargo alterations to PE, the specific molecular changes and their mechanistic roles in disease pathogenesis remain incompletely characterized.

As is well‐known, EVs are membrane‐bound nanoparticles that carry proteins, nucleic acids, and metabolites [12]. Intriguingly, we observed that NP‐EVs retained their ability to suppress CD4^+^ T cell inflammatory responses even after proteinase and RNase treatment. This finding prompted us to conduct comprehensive metabolomic profiling to characterize the metabolite cargo of these EVs. Nicotinamide (NAM), a water‐soluble vitamin B3 derivative, plays important roles as an anti‐inflammatory agent and essential precursor for nicotinamide adenine dinucleotide (NAD+) biosynthesis [13, 14]. Recent evidence suggests that exogenous NAM administration alleviates hypertension and endothelial dysfunction in PE mouse models, potentially through inhibition of ADP ribosyl cyclase activity [15]. However, the distribution and biological functions of NAM at the human maternal‐fetal interface remain to be elucidated. Our study shows that high concentrations of NAM enriched in pEVs are essential for regulating maternal‐fetal immune tolerance. The biogenesis of EVs involves complex cellular processes, beginning with endosomal membrane invagination and maturation into multivesicular bodies (MVBs) through fusion with Golgi‐derived vesicles [16]. And the endosomal sorting complex required for transport (ESCRT) machinery plays a pivotal role in cargo selection and vesicle formation, particularly protein and RNA [17]. However, the regulatory mechanisms underlying alterations in pEV metabolites remain unexplored.

In this study, we elucidated the transport pathways through which pEVs deliver NAM and the mechanism by which NAM in pEVs regulate maternal‐fetal immune homeostasis. These findings uncover a novel pathway implicated in the pathogenesis of PE and provide potential strategies for its therapeutic intervention.

## 2 Results

### 2.1 PE Patients Derived pEVs Induce Th1/Th2 Imbalance With Decreased NAM Levels Correlating With PE Severity Markers

We isolated human placental‐derived EVs (pEVs) through phycoerythrin‐conjugated placental alkaline phosphatase (PLAP) antibody [18]. NP and PE patients derived pEVs (huNP‐EVs and huPE‐EVs) were all positive for CD63, ALIX, and TSG101 molecules and rich in PLAP but were negative for the endoplasmic reticulum‐residing protein GRP94 (Figure S1A). Most of the pEVs were 40–200 nm in size, as determined by TEM (Figure S1B). Nanoparticle tracking analysis (NTA) revealed that the mean sizes of the pEVs from huNP and huPE patients were 153.9 and 148.7 nm (Figure S1C). Further study revealed that there was no significant difference in the amount of EVs secreted by placenta villus of the same weight from NP and PE patients (Figure S1D).

**FIGURE 1:**
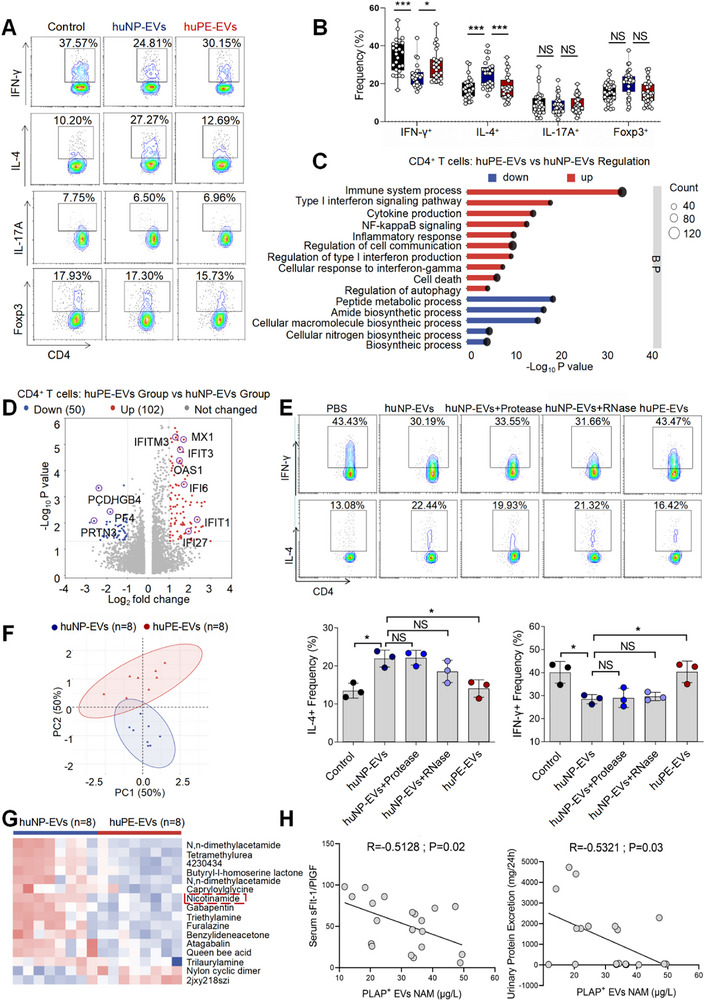
(A) Decreased NAM levels in pEVs correlates with Th1/Th2 cell imbalance and indicators of PE severity. Frequencies of IFN‐γ^+^ CD4^+^, IL‐4^+^ CD4^+^, IL‐17A^+^ CD4^+^, Foxp3^+^ CD4^+^ T cells were analyzed by flow cytometry. (B) Statistical analysis of the frequencies of CD4^+^ T cells. (C) GO enrichment terms that were significantly enriched in the differentially expressed genes in CD4^+^ T cells treated with NP‐EVs or PE‐EVs. (D) The volcano map shows a comparison of the content and *p* value of gene expression between CD4^+^ T cells treated with NP‐EVs or PE‐EVs. Differential genes were screened out by fold change greater than or equal to 2.0 and p < 0.05. (E) Frequencies of IFN‐γ^+^ CD4^+^, IL‐4^+^ CD4^+^ T cells were analyzed by flow cytometry. (F) Principal component analysis (PCA) reflected the differences between the two groups of pEVs (*n* = 8). (G) The heatmap shows differential metabolite expression between NP‐EVs and PE‐EVs. (H) A correlation analysis with pEV NAM levels and both the sFlt‐1/PlGF ratio and 24‐h proteinuria. Data normality was verified using Shapiro‐Wilk test. The dataset in figure (E) exhibited non‐normal distribution and was consequently analyzed using the Kruskal–Wallis test, the normally distributed data in figure (B) were evaluated by one‐way ANOVA. All data are presented as mean ± SEM (*p < 0.05, ***p < 0.001, NS, not significant).

In our previous study, we demonstrated that huPE‐EVs promote macrophage toward a CD86‐high pro‐inflammatory nature (pro‐inflam Macs) compared to huNP‐EVs [8] (Figure S1E,F). However, the regulatory mechanisms through which pEVs modulate CD4^+^ T cells remain to be elucidated. In this study, CD4^+^ T cells derived from peripheral blood were cocultured with huNP‐EVs and huPE‐EVs separately. We found that huNP‐EVs hindered IFN‐γ^+^ T cells differentiation and prompted the differentiation of Th2‐type cell, whereas their influence on Th17 and Treg cell subsets remained unimpressive (Figure 1A,B). RNA transcriptome was performed on CD4^+^ T cells treated with huNP‐EVs or huPE‐EVs (*n* = 10). Gene Ontology (GO) enrichment results showed that genes with elevated expression in the huPE‐EVs group were enriched in multiple immune responses and inflammation‐related biological processes, such as “inflammatory responses” and “NF‐kappaB signaling” (Figure 1C). Higher expression of OAS1, OAS2, MX1, MX2, IFIT1, IFITM3, IFI27, etc., was observed in CD4^+^ T cells treated with huPE‐EVs compared with NP‐EVs group (Figure 1D).

To investigate the functional components of huNP‐EVs in inhibiting CD4^+^ T cell inflammatory, we treated them with proteinase and RNase to degrade protein and RNA content, respectively. Following co‐culture with CD4^+^ T cells, we observed that the suppression of Th1 cell differentiation persisted despite protein and RNA degradation (Figure 1E). These findings suggest that EV‐associated metabolites may play a crucial role in mediating immunoregulatory functions. Untargeted metabolomics was conducted on huNP‐EVs and huPE‐EVs (*n* = 8), with the clinical characteristics of NP and PE patients detailed in Table 3. We used the VIP values of the first two principal components of the partial least squares method‐discriminatory analysis (PLS‐DA) model, combined with the results of Student's t test obtained by univariate analysis to screen the differential metabolites (Figure 1F). The levels of metabolites such as 2jxy218szi and nylon cyclic dimer were significantly elevated in huPE‐EVs, whereas the levels of metabolites such as nicotinamide (NAM), triethylamine and furalazine were significantly decreased in huPE‐EVs (Figure 1G).

A correlation analysis with the differential untargeted metabolism of pEVs and the transcriptome of 43 differentially expressed genes in CD4^+^ T cells treated with NP‐EVs or PE‐EVs associated with the GO‐enriched term “immune responses” was performed. NAM emerged as a pivotal player, occupying strategic positions within the metabolite regulatory network that governs alterations in the ‘immune response’‐associated genes of CD4^+^ T cells (Figure S1G). To further explore the clinical relevance of NAM levels in pEVs in PE progression, we analyzed the correlation between NAM content in patient‐derived pEVs and established clinical parameters, including the sFlt‐1/PlGF ratio in peripheral blood and 24‐h urinary protein excretion [19]. Our analysis revealed a significant negative correlation between pEV NAM levels and both the sFlt‐1/PlGF ratio (*r* = −0.5128, *p* = 0.02) and 24‐h proteinuria (*r* = −0.5321, *p* = 0.03) (Figure 1H).

These findings show that, compared with huNP‐EVs, huPE‐EVs induce Th1/Th2 imbalance, with NAM levels significantly decreased and strongly correlating with PE severity markers.

### 2.2 PE Mouse Derived pEVs' Impaired Capacity to Induce Maternal‐fetal Immune Tolerance Is Associated With PE‐Like Symptoms

**FIGURE 2:**
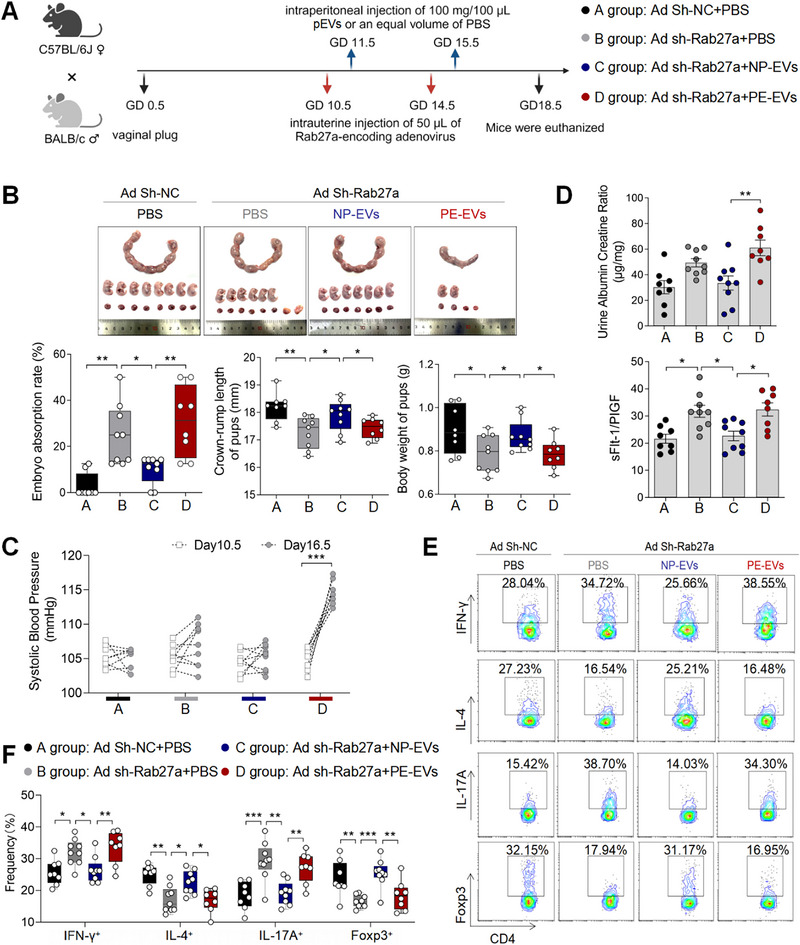
PE Mouse pEVs Disrupt Maternal‐fetal Immune Tolerance and Contribute to the Pathogenesis of PE. (A) Animal experimental model of intrauterine adenovirus infusion followed by pEVs intraperitoneal injection. (B) Embryo abortion rate of the pregnant mice, body weight and crown‐rump length of pups measured on day 18.5 of gestation. Black represents mice intrauterine infusion of negative control adenovirus accompanied by intraperitoneal injection of PBS (the number of pregnant mice: 8; the number of fetuses per pups of each pregnant mice: 8, 8, 6, 6, 8, 10, 7, 9); gray represents mice intrauterine infusion of Rab27a adenovirus accompanied by intraperitoneal injection of PBS (the number of pregnant mice: 9; the number of fetuses per pups of each pregnant mice: 7, 6, 6, 2, 6, 6, 5, 3, 6); blue represents mice intrauterine infusion of Rab27a adenovirus accompanied by intraperitoneal injection of mNP‐EVs (the number of pregnant mice: 9; the number of fetuses per pups of each pregnant mice: 9, 6, 8, 7, 7, 6, 6, 6, 7); red represents mice intrauterine infusion of Rab27a adenovirus accompanied by intraperitoneal injection of mPE‐EVs (the number of pregnant mice: 8; the number of fetuses per pups of each pregnant mice: 2, 2, 7, 5, 7, 6, 7, 6). (C) SBP of pregnant mice in the four groups. (D) UACR in urine and sFlt‐1/PlGF ratio in serum of pregnant mice in the four groups. (E) Frequencies of IFN‐γ^+^CD4^+^, IL‐4^+^CD4^+^, IL‐17A^+^CD4^+^, Foxp3^+^CD4^+^ T cells were analyzed by flow cytometry. (F) Statistical analysis of the frequencies of CD4^+^ T cells. Data normality was verified using Shapiro‐Wilk test. The dataset in figure (C) exhibited non‐normal distribution and was consequently analyzed using the Kruskal‐Wallis test, the normally distributed data in other figure were evaluated by one‐way ANOVA. All data are presented as mean ± SEM (*p < 0.05, **p < 0.01, ***p < 0.001, NS, not significant).

To investigate the contributory role of pEVs in the maintaining normal pregnancy in vivo, we established a pregnant mouse model by intrauterine infusion of Rab27a adenovirus to inhibit pEVs secretion (Figure 2A). Rab27a, a key regulator of multivesicular body (MVB)‐plasma membrane fusion, was knocked down, leading to impaired EV secretion [20]. Through intrauterine administration of Rab27a adenovirus at varying concentrations, we identified that a threshold of 10^11^ copies mL^−1^ resulted in a tendency of increased embryo absorption rate and impaired fetal growth (Figure S2A), as evidenced by tendencies of reduced crown‐rump length and fetal weight, while systolic blood pressure (SBP) remained unchanged (Figure S2B,C). At this concentration, placental Rab27a expression was reduced at both the protein and mRNA levels (Figure S2D,E), consequently leading to a decrease in placental EV release (Figure S2F). We also observed a shift towards anti‐inflammatory immune cells (Th1 cells and Th17 cells) at the maternal‐fetal interface in pregnant mice treated with Rab27a adenovirus (Figure 2E,F). However, the placental labyrinth zone (LZ) to trophoblast zone (TZ) ratio remained unchanged in the mouse model group (Figure S2G). Similarly, lentivirus‐mediated si‐Rab27A was used to knock down Rab27a in HTR‐8 cells, achieving ≈75% efficiency (Figure S2H,I); despite this efficient knockdown, no significant effect on the invasion or migration capacity of trophoblast cells was observed in vitro (Figure S2J,K).

We used Sham surgery and the Reduced Uterine Perfusion Pressure (RUPP) model to mimic normotensive NP and PE, respectively. In our 2025 *eLife* study, we demonstrated that the RUPP model effectively recapitulates PE [21]. We isolated mouse placental‐derived EVs (pEVs) through Phycoerythrin‐conjugated Cytokeratins7 (CK7) antibody (Figure S3A) [22]. Most of the pEVs were 40–200 nm in size, as determined by TEM (Figure S3B). NTA revealed that the mean sizes of the pEVs from NP and PE mouse were 163.6 and 166.9 nm, respectively (Figure S3C). And NAM level in PE‐EVs was significantly lower than NP‐EVs (Figure S3D). Tracking experiments revealed that intraperitoneally injected pEVs could accumulate in the mouse uterus (Figure S3E).

The NP‐EVs and PE‐EVs were injected to pregnant mice pre‐treated with Rab27a adenovirus. Compared with Ad Sh‐Rab27a group, mouse in NP‐EVs group induced lower embryo absorption rate, higher crown‐rump length and fetal weight (Figure 2B). However, compared with NP‐EVs, PE‐EVs fail to maintain pregnancy and maternal‐fetal immune balance. Instead, they lead to PE‐like symptoms such as elevated systolic blood pressure (SBP), urine albumin‐to‐creatinine ratio (UACR), and sFlt‐1/PlGF ratios (Figure 2C,D), while also inducing increased frequencies of Th1 cells and Th17 cells (Figure 2E,F).

Moreover, by administering GW4869 to pregnant mice—a compound that inhibits EV release by blocking ceramide‐mediated multivesicular budding [23]—we found that subsequent treatment with either NP‐EVs or PE‐EVs produced results consistent with those observed with Rab27a adenovirus (Figure S4).

These findings reveal the critical role of NP‐EVs in maintaining pregnancy and maternal‐fetal immune tolerance, notably suggesting that PE‐EVs’ capacity to induce maternal‐fetal immune tolerance was impaired and may play a significant role in the pathogenesis of PE in mouse model.

### 2.3 Decreased NAM Levels in pEVs Undermine the Ability to Induce Maternal‐Fetal Immune Tolerance and Exacerbate PE‐Like Symptoms

**FIGURE 3:**
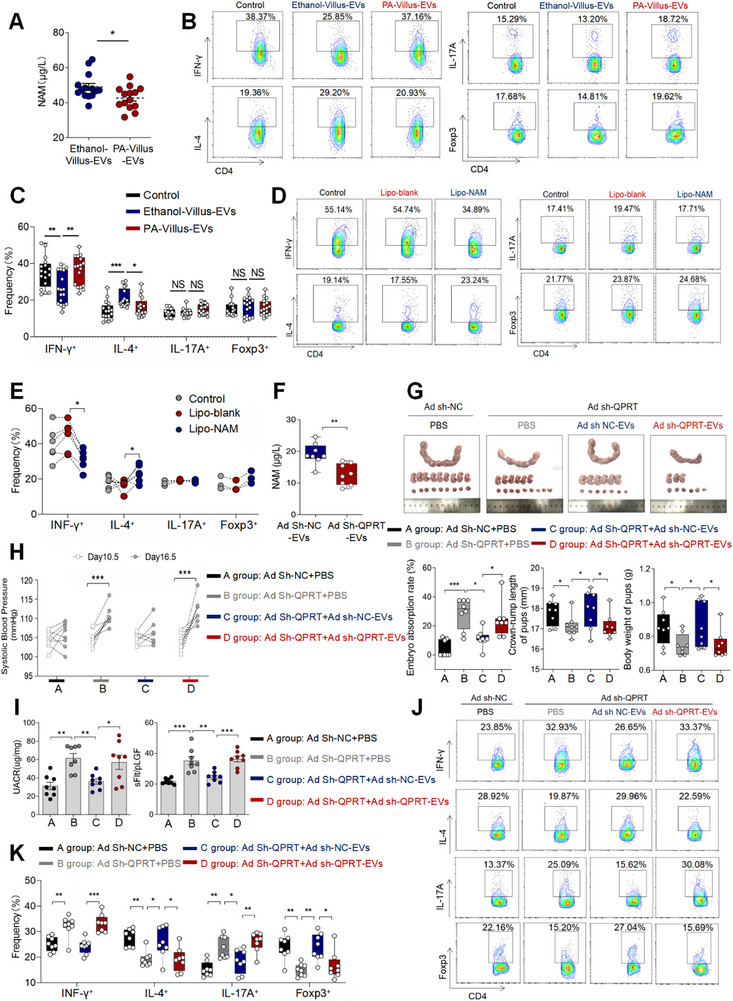
Decreased NAM Level in PE‐EVs Lead to the Maternal‐fetal Immune Dysregulation as Well as the Development of PE. (A) Concentration of NAM in Ethanol‐Villus‐EVs and PA‐Villus‐EVs (n=14). (B) The levels of IFN‐γ, IL‐4, Th17 and Treg in CD4^+^ T cells treated with Ethanol‐Villus‐EVs or PA‐Villus‐EVs. (C) Statistical analysis of the frequencies of Th1, Th2, Th17 and Treg cells. (D) The levels of IFN‐γ, IL‐4, Th17 and Treg in CD4^+^ T cells treated with PBS, Lipo‐blank or Lipo‐NAM. (E) Statistical analysis of the frequencies of IFN, IL‐17A, IL‐4 and Foxp3 in CD4^+^ T cells treated with PBS, Lipo‐blank or Lipo‐NAM. (F) Concentration of NAM in Ad sh‐NC‐EVs (n=8) and Ad sh‐*QPRT*‐EVs (n=8). (G) Embryo abortion rate of the pregnant mice, body weight and crown‐rump length of pups measured on day 18.5 of gestation. Black represents mice intrauterine infusion of negative control adenovirus accompanied by intraperitoneal injection of PBS (the number of pregnant mice: 8; the number of fetuses per pups of each pregnant mice: 8, 8, 8, 7, 7, 8, 8, 9); gray represents mice intrauterine infusion of *QPRT* adenovirus accompanied by intraperitoneal injection of PBS (the number of pregnant mice: 8; the number of fetuses per pups of each pregnant mice: 6, 5, 8, 5, 6, 7, 5, 5); blue represents mice intrauterine infusion of *QPRT* adenovirus accompanied by intraperitoneal injection of Ad Sh‐NC‐EVs (the number of pregnant mice: 8; the number of fetuses per pups of each pregnant mice: 12, 9, 7, 7, 6, 8, 7, 7); red represents mice intrauterine infusion of *QPRT* adenovirus accompanied by intraperitoneal injection of Ad Sh‐*QPRT* EVs (the number of pregnant mice: 8; the number of fetuses per pups of each pregnant mice: 7, 6, 3, 7, 6, 7, 7, 6). (H) SBP of pregnant mice in the four groups. (I) UACR in urine and sFlt‐1 vs PlGF in serum of pregnant mice in the four groups. (J) Frequencies of IFN‐γ^+^CD4^+^, IL‐4^+^CD4^+^, IL‐17A^+^CD4^+^, Foxp3^+^CD4^+^ T cells were analyzed by flow cytometry. (K) Statistical analysis of the frequencies of Th1, Th2, Th17 and Treg cells. Data normality was verified using Shapiro‐Wilk test. The dataset in figure (I)’ UACR, figure (k)’ IFN‐γ and IL‐4 exhibited non‐normal distribution and was consequently analyzed using the Kruskal–Wallis test. The normally distributed data in other figures were evaluated by Student's *t*‐test (two groups) or one‐way ANOVA (multiple groups). All data are presented as mean ± SEM (*p < 0.05, **p < 0.01, ***p < 0.001).

Quinolinate phosphoribosyltransferase (*QPRT*), a key enzyme in the de novo biosynthesis of NAD+, demonstrates a relationship between its activity and intracellular NAM levels [25]. Genetic knockdown or pharmacological inhibition of *QPRT* results in significantly reduced intracellular NAM concentrations [24]. To investigate the significance of NAM in pEVs, phthalic acid (PA), an inhibitor of QPRT [26], was applied to human early pregnancy villus explants to isolate EVs derived from PA‐treated villus explants (PA‐Villus‐EVs). We observed significantly lower NAM levels in PA‐Villus‐EVs compared to EVs isolated from villus explants treated with 5% ethanol (Ethanol‐Villus‐EVs) (Figure 3A). CD4^+^ T cells were treated with PBS, Ethanol‐Villus‐EVs or PA‐Villus‐EVs. We observed that Ethanol‐Villus‐EVs suppressed Th1 cell differentiation while promoting Th2 cell differentiation compared to PBS (control group), whereas PA‐Villus‐EVs had no effect. Neither of them had significant effects on the differentiation of Th17 or Treg cells (Figure 3B,C). To eliminate potential interference from other EV components on the results, we utilized a strategy of encapsulating NAM or PBS within liposomes (Lipo‐blank and Lipo‐NAM) to mimic extracellular vesicles. We firstly characterized Lipo blank and Lipo NAM using nanoparticle tracking analysis (NTA) and transmission electron microscopy (TEM) (Figure S5A,B). And the NAM loading efficiency in liposomes was 53.67±3.11% (Figure S5C). Our findings revealed that Lipo‐NAM could inhibit Th1 cell differentiation while promote Th2 cell differentiation, compared to PBS‐laden liposomes (Lipo‐blank) (Figure 3D,E). However, liposomal NAM did not significantly affect Th17/Treg cell differentiation (Figure 3D,E).

Subsequently, Ad sh‐NC EVs (control) or Ad sh‐*QPRT* EVs were generated by uterine perfusion with a NC‐ or *QPRT*‐targeting adenovirus to pregnant mice. NAM level in Ad sh‐*QPRT* EVs was significantly lower than Ad sh‐NC EVs (Figure 3F). Then, Pregnant mice were then injected with either Ad sh‐NC‐EVs or Ad sh‐*QPRT*‐EVs. Our results demonstrated that Ad sh‐NC‐EVs improved pregnancy outcomes in *QPRT*‐targeting adenovirus uterine perfusion mice model. In contrast, Ad sh‐*QPRT* EVs treatment resulted in significantly increased embryo resorption rates, reduced crown‐rump length, and decreased fetal weight compared to the Ad sh‐NC‐EVs group (Figure 3G). Notably, mice treated with Ad sh‐*QPRT*‐EVs exhibited PE‐like symptoms, including elevated systolic blood pressure (SBP) and urine albumin‐to‐creatinine ratio (UACR), along with altered sFlt‐1/PlGF ratios (Figure 3H,I). Furthermore, in the *QPRT*‐targeting adenovirus‐induced PE mouse model, we observed increased frequencies of Th1 cells and Th17 cells, accompanied by decreased frequencies of Th2 cells and Treg cells (Figure 3J,K).

To validate the role of NAM in improving pregnancy outcomes in PA‐induced PE mouse models, we exogenously supplemented NAM in pregnant mice. First, uterine perfusion of 50 mg/kg PA was found to increase the embryo absorption rate, reduce crown‐rump length and fetal weight compared with control group (Figure S6A), and elevate blood pressure and UACR in pregnant mice (Figure S6B). Interestingly, when PA‐treated mice were administered PE‐EVs, PE‐like symptoms persisted, along with sustained increased frequencies of Th1 and Th17 cells at the maternal‐feta interface (Figure S6C,D). However, when NAM was injected into those PE‐EVs‐treated mice, we observed a reduction in the embryo absorption rate, as well as increased crown‐rump length and body weight of pups (Figure S6A). Additionally, SBP and UACR were significantly decreased (Figure S6B). NAM was also shown to significantly inhibit Th1 cell and Th17 cell differentiation (Figure S6C,D).

These findings highlight that decreased NAM levels in PE‐EVs correlate with impaired capacity to induce maternal‐fetal immune tolerance and exacerbated PE‐like symptoms.

### 2.4 NAM in pEVs Inhibits Th17 Cell Differentiation via Macrophages

**FIGURE 4:**
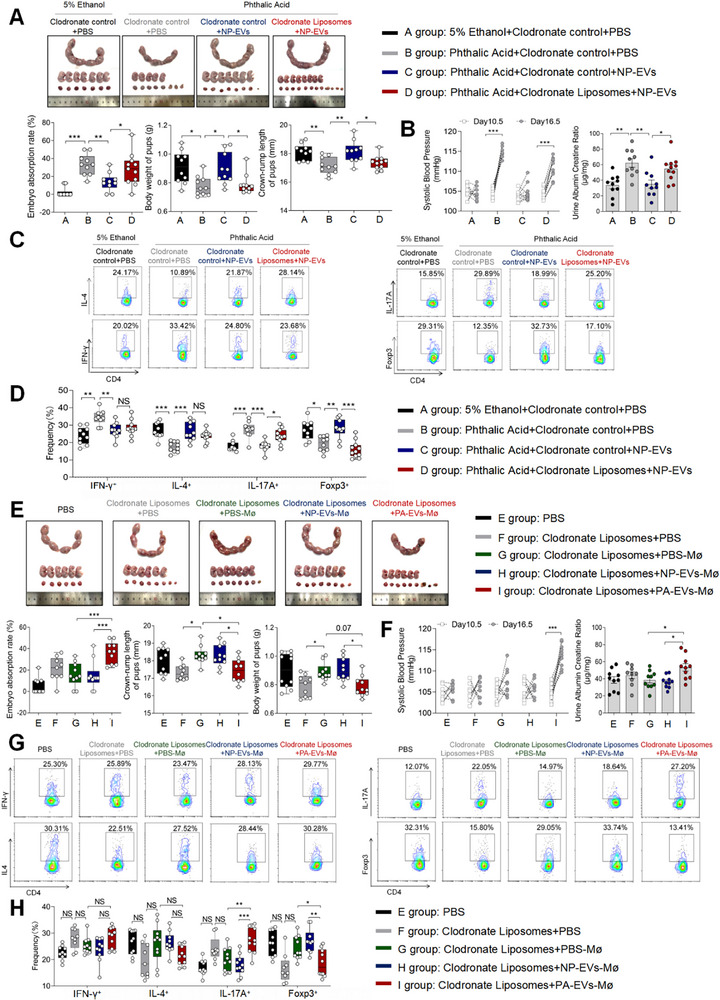
NAM in pEVs Promote Pro‐inflammatory Macrophages Thereby Induce Th17 Cell Differentiation. (A) Embryo abortion rate of the pregnant mice, body weight and crown‐rump length of pups measured on day 18.5 of gestation. Black represents mice intrauterine infusion of 5% ethanol accompanied by intraperitoneal injection of PBS (the number of pregnant mice: 10; the number of fetuses per pups of each pregnant mice: 7, 7, 9, 7, 8, 8, 1, 7, 9, 7); gray represents mice intrauterine infusion of PA accompanied by intraperitoneal injection of PBS (the number of pregnant mice: 10; the number of fetuses per pups of each pregnant mice: 5, 6, 4, 7, 6, 2, 7, 2, 7, 3); blue represents mice intrauterine infusion of PA accompanied by intraperitoneal injection of NP‐EVs (the number of pregnant mice: 10; the number of fetuses per pups of each pregnant mice: 2, 7, 9, 3, 7, 5, 5, 6, 6, 6); red represents mice intrauterine infusion of PA accompanied by intraperitoneal injection of NP‐EVs after injection of clodronate liposomes (the number of pregnant mice: 11; the number of fetuses per pups of each pregnant mice: 5, 8, 6, 8, 5, 2, 4, 9, 6, 5, 7). (B) SBP and UACR of pregnant mice in the four groups. (C) Frequencies of IFN‐γ^+^CD4^+^, IL‐4^+^CD4^+^, IL‐17A^+^CD4^+^, Foxp3^+^CD4^+^ T cells were analyzed by flow cytometry. (D) Statistical analysis of the frequencies of Th1, Th2, Th17 and Treg cells. (E) Embryo abortion rate of the pregnant mice, body weight and crown‐rump length of pups measured on day 18.5 of gestation. Black represents mice intraperitoneal injection of PBS (the number of pregnant mice: 10; the number of fetuses per pups of each pregnant mice: 6, 7, 8, 9, 8, 2, 11, 9, 9, 9); gray represents mice intraperitoneal injection of clodronate liposomes (the number of pregnant mice: 9; the number of fetuses per pups of each pregnant mice: 8, 5, 6, 6, 5, 7, 6, 6, 6); green represents mice injected PBS‐treated BMDMs after intraperitoneal injection of clodronate liposomes (the number of pregnant mice: 10; the number of fetuses per pups of each pregnant mice:10, 4, 6, 6, 7, 6, 7, 2, 5, 8); blue represents mice injected mNP‐EVs‐treated BMDMs after intraperitoneal injection of clodronate liposomes (the number of pregnant mice: 10; the number of fetuses per pups of each pregnant mice: 7, 4, 8, 6, 7, 7, 1, 7, 4, 8); red represents mice injected mPA‐EVs‐treated BMDMs after intraperitoneal injection of clodronate liposomes (the number of pregnant mice: 10; the number of fetuses per pups of each pregnant mice: 6, 6, 5, 4, 5, 7, 2, 2, 4, 3). (F) SBP and UACR of pregnant mice in the five groups. (G) Frequencies of IFN‐γ^+^CD4^+^, IL‐4^+^CD4^+^, IL‐17A^+^CD4^+^, Foxp3^+^CD4^+^ T cells were analyzed by flow cytometry. (H) Statistical analysis of the frequencies of IFN‐γ^+^CD4^+^, IL‐4^+^CD4^+^, IL‐17A^+^CD4^+^, Foxp3^+^CD4^+^ T cells. Data normality was verified using Shapiro–Wilk test. The dataset in figure (A)′ body weight of pups, figure (E)′ Embryo abortion rate of the pregnant mice and body weight of pups exhibited non‐normal distribution and was consequently analyzed using the Kruskal–Wallis test. The normally distributed data in other figure were evaluated by Student's *t*‐test (two groups) or one‐way ANOVA (multiple groups). All data are presented as mean ± SEM (*p < 0.05, **p < 0.01, ***p < 0.001).

We found that NAM within pEVs had no significant regulatory effect on the Th17/Treg balance in vitro but could inhibit Th17 cell differentiation in vivo. Notably, our research group's 2025 eLife publication demonstrated that pro‐inflammatory macrophages promote Th17 cell differentiation at the maternal‐fetal interface [21]. PBS or NP‐EVs were administered to pregnant mice following intrauterine perfusion with PA. Compared to PBS, NP‐EVs alleviated PE‐like symptoms (Figure 4A,B) and restored maternal‐fetal immune balance, as evidenced by reduced frequencies of Th1 cells, Th17 cells (Figure 4C,D). However, depleting macrophages with clodronate liposomes [21] before injecting NP‐EVs abolished the therapeutic effects of NP‐EVs. Specifically, the inhibitory effect of NP‐EVs on Th17 cell differentiation at the maternal‐fetal interface in PA‐treated mice was eliminated upon macrophage depletion, while the regulation of Th1/Th2 balance and macrophage polarization remained unaffected (Figure 4C,D).

To further confirm that pEVs modulate the Th17/Treg balance through macrophage regulation, PA treated mouse placental derived EVs (PA‐EVs)‐ or NP‐EVs‐treated Bone Marrow‐Derived Macrophages (BMDMs) were transfused into pregnant mice pretreated with clodronate liposomes. A significantly higher embryo resorption rate, along with reduced crown‐rump length and body weight of pups, was observed in the PA‐EVs‐BMDMs group compared to the NP‐EVs‐BMDMs group (Figure 4E). SBP and UACR levels during gestation were also markedly elevated in the PA‐EVs‐BMDMs group (Figure 4F). Flow cytometry analysis revealed a significant increase in the frequency of Th17 cells and a concurrent decrease in the frequency of Treg cells at the maternal‐fetal interface in the PA‐EVs‐BMDMs group, while the Th1/Th2 balance remained unchanged (Figure 4G,H).

LPS is a well‐established and potent immunostimulant that robustly activates macrophages and induces a pro‐inflammatory state [27, 28]. To investigate the mechanisms underlying NAM's inhibition of pro‐inflam Macs polarization, RNA transcriptome was performed to analyze the mRNA landscape in LPS treated‐BMDMs after cocultured with Ad s‐ NC‐EVs or Ad sh‐*QPRT*‐EVs. The results revealed that macrophages cocultured with Ad Sh‐*QPRT*‐EVs exhibited upregulated expression of genes associated with inflammatory factors and chemokines, such as *Il1β*, *Tnf‐α*, *Cxcl1*, and *Cxcl5*, compared to those treated with Ad Sh‐NC‐EVs (Figure S7A). GO enrichment analysis indicated that genes upregulated in the Ad Sh‐*QPRT*‐EVs group were primarily involved in “inflammatory responses,” while downregulated genes were enriched in “ATP‐dependent activities,” suggesting impaired oxidative phosphorylation in macrophages (Figure S7B). A decrease in NAD+/NADH levels and the expression of oxidative phosphorylation‐related genes (e.g., *Atp5j2, Cox7c, Ndufb7, Ndufb9*) was observed in Ad Sh‐*QPRT*‐EVs‐treated macrophages compared to the Ad Sh‐NC‐EVs group (Figure S7C,D). We also found a reduction in oxidative phosphorylation (OxPhos) capacity in the Ad Sh‐*QPRT*‐EVs group, as demonstrated by significantly decreased maximal, ATP‐related, and alternative respiratory capacities (Figure S7E). Conversely, an increase in glycolytic capacity was observed, indicated by elevated glycolysis, glycolytic capacity, and glycolytic reserve levels (Figure S7F). Notably, previous studies have reported that such metabolic shifts are closely associated with M1‐like pro‐inflam Macs polarization [29].

These results demonstrate the critical role of pEVs in maintaining Th17/Treg homeostasis through macrophage regulation.

### 2.5 SIRT1 Activators Abolish the Suppression of Th1 Differentiation but Not Inhibition of Inflam Macs Induced by NAM of pEVs

Given that pEVs can modulate both Th1/Th2 and Th17/Treg cell balance at the maternal‐fetal interface, we sought to determine which immunoregulatory axis plays a more dominant role in pEV‐induced PE. To investigate this, we conducted in vivo neutralization experiments targeting IFN‐γ (primarily produced by Th1 cells) and IL‐17A (mainly secreted by Th17 cells). Our results demonstrated that IFN‐γ neutralization significantly improved pregnancy outcomes compared to the PE‐EVs‐only group, as evidenced by reduced embryo resorption rates, increased crown‐rump length, and higher fetal weight (Figure S8A). Anti‐IFN‐γ therapy Also significantly lowered SBP and reduced the UACR compared to the PE‐EVs control group (Figure S8B). Although IL‐17A neutralization also improved some PE parameters, such as increasing fetal weigh and crown‐rump length, it was overall inferior to IFN‐γ antibody treatment (Figure S8A,B), suggests Th1 cells may play a more central role in pEVs‐induced pathogenesis than Th17 cells.

**FIGURE 5:**
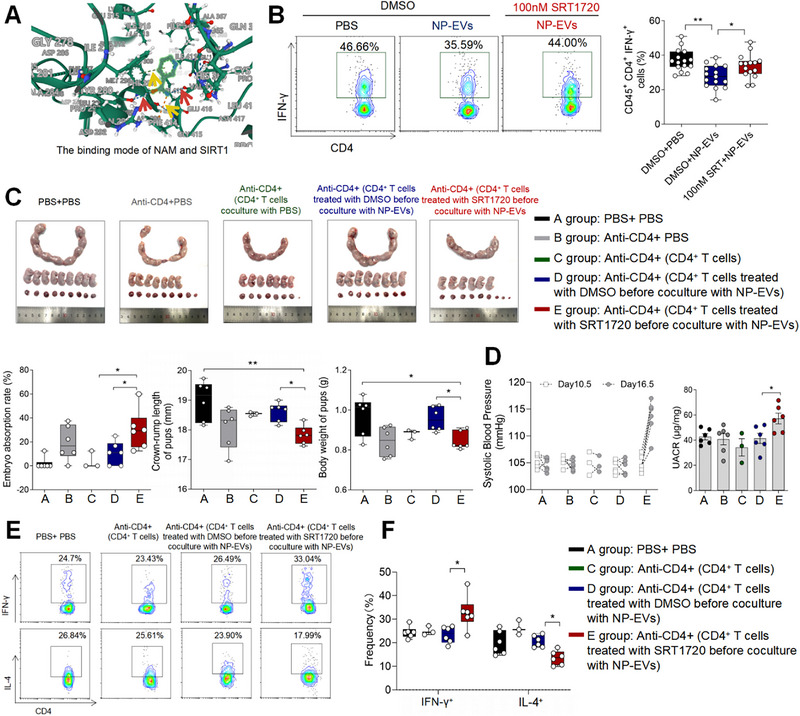
NAM in pEVs Suppresses Th1 Differentiation by Inhibiting SIRT1 in CD4^+^ T Cells. (A) Crystal structure of NAM bound to SIRT1.Yellow arrows indicate aromatic ring bonds and red arrows indicate hydrogen bonds. (B) Frequencies of IFN‐γ^+^ CD4^+^ T cells were determined by flow cytometry. (C) Picture of mouse uterus at 18.5 days of gestation of each group. Black represents negative control (the number of pregnant mice: 6; the number of fetuses per pups of each pregnant mice: 7, 4, 3, 9, 7, 9); gray represents mice treated with anti‐CD4 antibody accompanied by intraperitoneal injection of PBS (the number of pregnant mice: 6; the number of fetuses per pups of each pregnant mice: 7, 8, 3, 5, 6, 8); green represents mice treated with anti‐CD4 antibody accompanied by CD4^+^T cells coculture with PBS (the number of pregnant mice: 3; the number of fetuses per pups of each pregnant mice: 8, 8, 7); blue represents mice treated with anti‐CD4 antibody accompanied by CD4^+^T cells coculture with NP‐EVs (the number of pregnant mice: 6; the number of fetuses per pups of each pregnant mice: 6, 8, 4, 7, 8, 8); red represents mice treated with anti‐CD4 antibody accompanied by CD4^+^T cells coculture with SRT1720 before NP‐EVs (the number of pregnant mice: 6; the number of fetuses per pups of each pregnant mice: 6, 2, 5, 5, 7, 7). Embryo abortion rate of female C57 mice, body weight and crown‐rump length of pups were measured on day 18.5 of gestation. (D) SBP and UACR of pregnant mice in the five groups. (E) The frequencies of IFN‐γ^+^ CD4^+^ T cells and IL‐4^+^ CD4^+^ T cells were determined by flow cytometry. (F) Statistical analysis of the frequencies of CD4^+^ T cells. Data normality was verified using Shapiro–Wilk test. The dataset in figure (C)′ Embryo abortion rate and body weight of pups exhibited non‐normal distribution and was consequently analyzed using the Kruskal–Wallis test, the normally distributed data in other figures were evaluated by one‐way ANOVA. All data are presented as mean ± SEM (*p < 0.05, **p < 0.01, NS, not significant).

The NAD‐dependent deacetylase Sirtuin 1 (SIRT1) plays a crucial role in regulating IFN‐γ production [30]. NAM, the primary NAD+ precursor and a noncompetitive inhibitor of SIRT1 [31], was investigated for its interaction with SIRT1. The structures of NAM and SIRT1 were obtained from the PubChem (http://pubchem.ncbi.nlm.nih.gov/) and RCSB PDB (http://www.rcsb.org/) databases, respectively, and their interaction was visualized using AutodockVina 1.2.2 (Figure 5A). First, our study demonstrated no correlation between SIRT1 activity in trophoblast cell lines (HTR‐8/SVneo and JEG‐3) and NAM content in pEVs. Pharmacological activation of SIRT1 using selisistat (EX‐527) failed to modulate EV‐associated NAM levels in these trophoblast models (Figure S9A). Furthermore, placental SIRT1 expression showed no significant difference between NP and PE patients (Figure S9B). Then, we found that when SIRT1 in CD4^+^ T cells was activated by SRT1720 [32] prior to coculture with pEVs, a significant increase in the frequency of IFN‐γ^+^CD4^+^ T cells was observed compared to DMSO‐treated cells (Figure 5B). Subsequently, CD4^+^ T cells from non‐pregnant C57 mice were treated with SRT1720 or DMSO and cocultured with NP‐EVs. These cells were then injected into anti‐CD4 antibody‐treated pregnant C57 mice. Mice injected with SRT1720‐treated CD4^+^ T cells exhibited higher embryo resorption rates, reduced fetal weights, and shorter crown‐rump lengths compared to controls (Figure 5C,D). Furthermore, mice receiving SRT1720‐pretreated CD4^+^ T cells showed elevated SBP and UACR levels compared to those receiving DMSO‐pretreated CD4^+^ T cells (Figure 5E,F). At the maternal‐fetal interface, the SRT1720 group displayed an increased frequency of IFN‐γ^+^CD4^+^ Th1 cells and a decreased frequency of IL‐4^+^CD4^+^ T cells (Figure 5G,H). These in vivo experiments confirmed that SIRT1 activation in CD4^+^ T cells counteracts the ability of NP‐EVs to alleviate PE symptoms.

To investigate whether NAM in EVs also regulates macrophage polarization through SIRT1, bone marrow‐derived macrophages (BMDMs) were pretreated with DMSO or SRT1720 before coculture with NP‐EVs. We found that SRT1720 treatment did not inhibit the polarization of macrophages toward an M2‐like phenotype induced by NP‐EVs (Figure S9C,D).

In conclusion, these results suggest that NAM in pEVs exerts its anti‐inflammatory effects may through inhibiting SIRT1 in CD4^+^ T cells.

### 2.6 The Decreased NAM Levels in PE Patients pEVs Result From Reduced HRS in Trophoblasts

**FIGURE 6:**
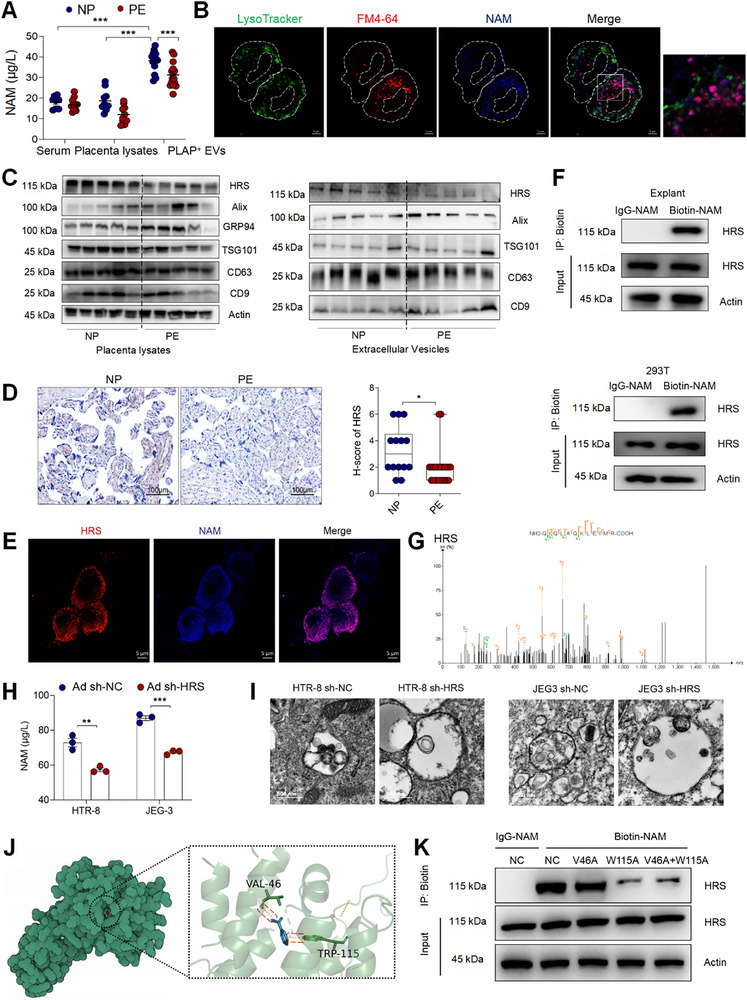
NAM Is Selectively Packaged Into EVs Through an HRS‐dependent Mechanism. (A) The concentration of NAM in serum, placenta lysates and pEVs from women with NP and PE was detected by ELISA. (B) Images of JEG3 cells stained with FM4‐64 (red), LysoTracker (green) and NAM (blue) were observed by confocal. (C) The protein expression of ESCRT in placenta lysates and EVs from women with NP and PE were analyzed. D) Immunochemistry images of HRS expression in the placental tissues of women with NP and PE. Scale bar = 100 µm. (E) Images of 293T cells stained with anti‐HRS (red) and NAM (blue) were observed by confocal. (F) Western blot analyses of whole‐cell lysates (input) and immunoprecipitates (IP) from explants and 293T cells upon NAM treatment for the indicated time points. G) Mass spectrometry analysis of the different proteins in placenta explants treated with con‐NAM and NAM‐biotin. Scale bars, 10 µm. (H) The level of NAM in EVs isolated from HRS knockdown (sh‐HRS) or negative control (sh‐NC) HTR‐8 cells and JRG3 cells was detected by ELASA. (I) Electron microscopy images showing the morphology change with MVBs in HTR‐8 cells and JRG3 cells treated with Ad Sh‐NC or Ad Sh‐HRS. (J) Crystal structure of NAM bound to HRS. (K) Western blot analysis of input and IP from 293T cells transfected with point mutations at the three candidate binding sites. Data normality was verified using Shapiro‐Wilk test. The dataset in these figures exhibited normal distribution and was consequently analyzed using the Student's *t*‐test. All data are presented as mean ± SEM (*p < 0.05, **p < 0.01, ***p < 0.001, NS, not significant).

To investigate the mechanisms underlying the diminished NAM levels in PE‐EVs, we collected and measured NAM levels in peripheral blood serum, placental lysates, and pEVs from both NP and PE patients. No significant differences were observed in NAM levels in blood serum or placental lysates between NP and PE patients. However, the concentration of NAM in PE‐EVs was significantly lower than in NP‐EVs, while pEVs exhibited significantly higher NAM levels compared to placental lysates and blood serum (Figure 6A). To trace the distribution of NAM in trophoblasts, we used 7‐amino‐4‐methylcoumarin (AMC)‐conjugated NAM. We observed nearly complete overlap between NAM and FM4‐64, a fluorescent probe for endosomes, but no overlap with LysoTracker, a marker for lysosomes, indicating that NAM is associated with endosomes, where EVs are generated (Figure 6B).

To further explore the molecular machinery governing EV cargo selection, we focused on the endosomal sorting complex required for transport (ESCRT), a classical pathway involved in EV generation and cargo sorting. This complex includes essential proteins such as HRS, TSG101 and Alix, which are critical for the selective packaging of cargo into EVs [16]. Analysis of placental lysates and pEVs revealed decreased HRS expression in PE patients, while levels of other ESCRT proteins remained unchanged (Figure 6C,D). We also observed significant overlap in the expression patterns of HRS and NAM (Figure 6E). Immunoprecipitation confirmed that HRS binds to Biotin‐conjugated NAM in HEK‐293T cell line and villus explants (Figure 6F).

Additionally, we performed proteomic mass spectrometry on three samples each from the IgG‐NAM and Biotin‐NAM groups. We first identified protein set A, which was common across all three IgG‐NAM samples (Figure S10A), and protein set B, which was common across all three Biotin‐NAM samples (Figure S10B). We identified 447 proteins unique to set B but absent in set A (Figure S10C). HRS was identified as a unique protein present exclusively in the Biotin‐NAM group (Figure 6G; Figure S10D,E).

To determine whether HRS plays a critical role in NAM loading into EVs in trophoblasts, we used lentiviral‐mediated knockdown of HRS in HTR8/SVneo and JEG3 cells (Figure S10F–H). We observed a significant decrease in NAM levels within EVs in the sh‐HRS group (Figure 6H). Furthermore, HRS knockdown led to pronounced alterations in the morphology of intracellular multivesicular bodies (MVBs) in both HTR‐8/SVNEO and JEG3 cells, including increased vesicle size (Figure 6I). These findings suggest that HRS plays a crucial role in regulating MVB biogenesis and vesicle trafficking, ultimately influencing EV composition.

To investigate interactions between NAM and specific residues, we performed molecular docking using Autodock Vina 1.2.2. The results pinpointed two putative binding residues: valine 46 (V46) and tryptophan‐115 (W115) (Figure 6J). Both sites reside within the HRS VHS domain (amino acids 1–140), which is known to be essential for extracellular vesicle cargo sorting [33]. Since mutation of key residues to alanine (A) can produce loss‐of‐function effects [34], we generated alanine substitutions and performed immunoprecipitation. These assays demonstrated that the alanine substituted W115 mutation (W115A) impaired NAM–HRS binding than the alanine substituted V46 mutation (V46A) V46A mutation, indicating that W115 plays a critical role in mediating the transport of NAM by HRS (Figure 6K).

These findings establish a critical role of HRS in trophoblasts for mediating NAM packaging into EVs.

### 2.7 HRS Deficiency in Mouse Trophoblasts Impairs NAM Enrichment in pEVs, Disrupts Maternal‐Fetal Immune Homeostasis, and Causes Preeclampsia

**FIGURE 7:**
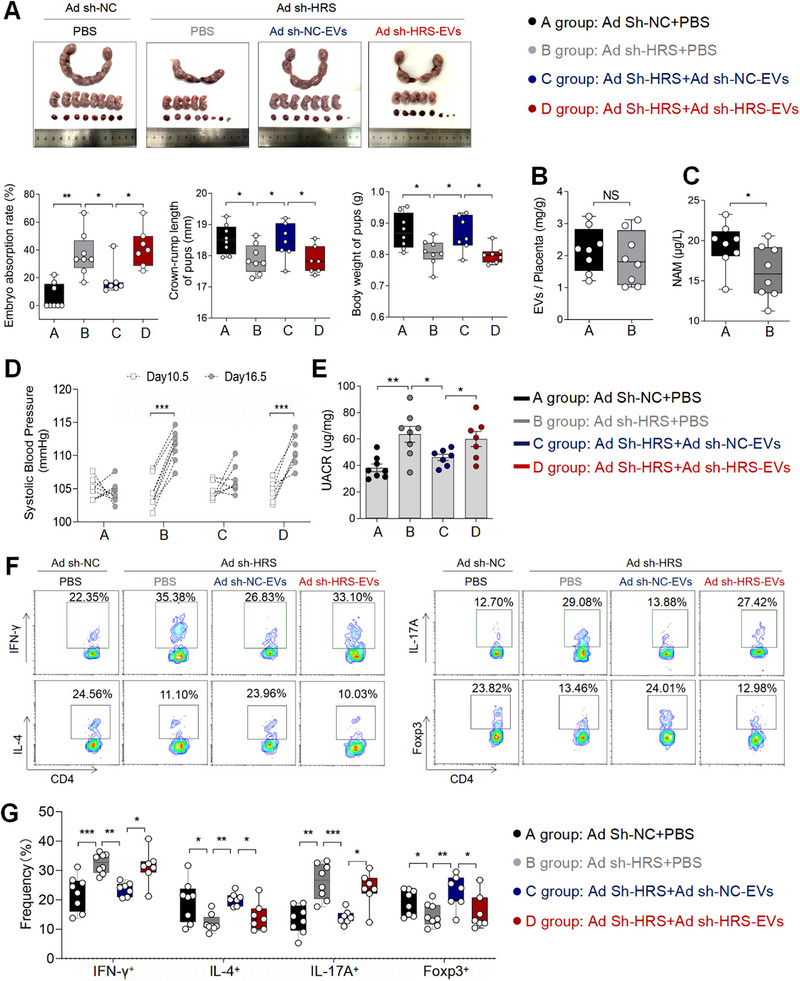
HRS Deficiency in trophoblasts reduced NAM Levels in pEVs and caused PE symptoms and maternal‐fetal immune imbalance. (A) Embryo abortion rate of the pregnant mice, body weight and crown‐rump length of pups measured on day 18.5 of gestation. Black represents mice intrauterine infusion of negative control adenovirus accompanied by intraperitoneal injection of PBS (the number of pregnant mice: 8; the number of fetuses per pups of each pregnant mice: 10, 7, 8, 8, 7, 7, 7, 5); gray represents mice intrauterine infusion of HRS adenovirus accompanied by intraperitoneal injection of PBS (the number of pregnant mice: 8; the number of fetuses per pups of each pregnant mice: 6, 5, 4, 5, 1, 2, 4, 1); blue represents mice intrauterine infusion of HRS adenovirus accompanied by intraperitoneal injection of Ad Sh‐NC‐EVs (the number of pregnant mice: 7; the number of fetuses per pups of each pregnant mice: 6, 5, 5, 8, 6, 7, 4); red represents mice intrauterine infusion of HRS adenovirus accompanied by intraperitoneal injection of Ad Sh‐HRS EVs (the number of pregnant mice: 7; the number of fetuses per pups of each pregnant mice: 6, 5, 1, 2, 3, 5, 5). (B) EVs secretion in placentas of mouse from Ad Sh‐NC group and Ad Sh‐HRS group. (C) Concentration of NAM in Ad sh‐NC‐EVs and Ad sh‐HRS‐EVs. (D) SBP of pregnant mice in the four groups. (E) UACR of pregnant mice in the four groups. (F) Frequencies of IFN‐γ^+^CD4^+^, IL‐4^+^CD4^+^, IL‐17A^+^CD4^+^, Foxp3^+^CD4^+^ T cells were analyzed by flow cytometry. (G) Statistical analysis of the frequencies of Th1, Th2, Th17, and Treg cells. Data normality was verified using Shapiro–Wilk test. The dataset in figure (E)′ UACR, figure (F)′ IFN‐γ and IL‐4 exhibited non‐normal distribution and was consequently analyzed using the Kruskal–Wallis test. The normally distributed data in other figures were evaluated by Student's *t*‐test (two groups) or one‐way ANOVA (multiple groups). All data are presented as mean ± SEM (*p < 0.05, **p < 0.01, ***p < 0.001).

To investigate the contributory role of HRS in loading NAM to pEVs in vivo, we established a pregnant mouse model by intrauterine infusion of Negative control (NC) or HRS adenovirus (Ad sh‐NC / Ad sh‐HRS). Compared with Ad Sh‐NC group, mouse in Ad sh‐HRS group induced higher embryo absorption rate, lower crown‐rump length and fetal weight (Figure 7A). Further analysis revealed that while placental EV secretion was comparable between two groups (Figure 7B), the NAM level in pEVs from Ad sh‐HRS group mouse (Ad sh‐HRS‐EVs) was significantly reduced compared to the Ad sh‐NC group mouse (Ad sh‐NC‐EVs) (Figure 7C). Then, we found that compared with Ad sh‐NC, Ad sh‐HRS group lead to PE‐like symptoms such as elevated SBP and UACR (Figure 7D,E), as well as maternal‐fetal immune balance characterized by increased frequencies of Th1 cells and Th17 cells (Figure 7F,G).

Furthermore, a decreased placental labyrinth zone‐to‐trophoblast zone (LZ/TZ) ratio was observed in the Ad sh‐HRS group mouse compared to the Ad sh‐NC group mouse (Figure S11A). This structural alteration is indicative of abnormal placental development, likely reflecting underlying imbalances in trophoblast differentiation and invasion. Lentivirus‐mediated si‐HRS was used to knock down HRS in HTR‐8 cells, achieving ≈85% efficiency (Figure S11B,C). Despite this efficient knockdown, no significant effect on the invasion or migration capacity of HTR‐8 cells was observed in vitro (Figure S11D,E). However, we found that supernatant from CD4+ T cells treated with NAM‐deficient pEVs (sh HRS‐EVs) suppressed trophoblast invasion compared to supernatant from CD4+ T cells treated with control pEVs (sh NC‐EVs) (Figure S11F,G). These results suggest that HRS deficiency impairs trophoblast invasion in vivo indirectly, likely by altering the cytokine profile secreted by CD4^+^ T cells in a pEV‐dependent manner.

Then we injected Ad sh‐NC‐EVs or A sh‐HRS‐EVs to Ad sh‐HRS group mouse, and found that compared with Ad sh‐NC‐EVs, Ad sh‐HRS‐EVs fail to maintain pregnancy characterized by higher embryo absorption rate, lower crown‐rump length and fetal weight (Figure 7A). Instead, they lead to PE‐like symptoms such as elevated SBP and UACR (Figure 7D,E), while also inducing increased frequencies of Th1 cells and Th17 cells (Figure 7F,G).

In conclusion, this study demonstrates that HRS deficiency in mouse trophoblasts impairs NAM enrichment in pEVs, which disrupts immune balance at the maternal‐fetal interface and leads to the development of PE.

### 2.8 The Degradation of HRS in Trophoblasts of PE Patients Is Triggered by Increased HSP27‐Mediated Ubiquitination

**FIGURE 8:**
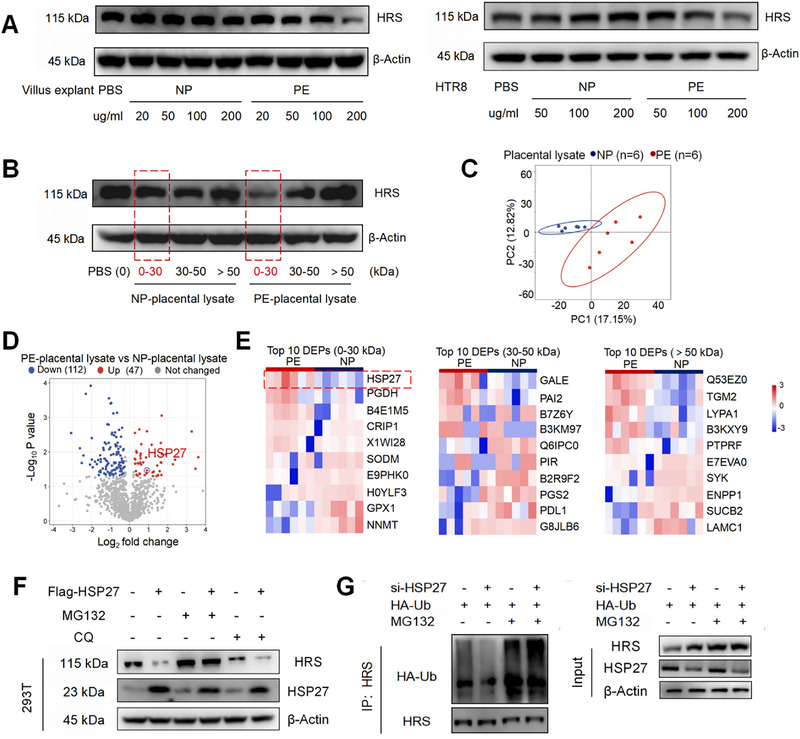
High Expression of HSP27 in PE Patients Placentas Promotes Ubiquitination‐mediated Degradation of HRS. (A) Western blot analyses HRS expression levels in HTR‐8/SVNEO cells treated with NP or PE placental villus explants lysate. (B) Western blot was performed to analyze HRS expression in JEG3 cells treated with proteins of varying molecular weights derived from NP and PE placental villus explants. (C) PCA reflected the differences between the two groups of pEVs (*n* = 6). (D) The volcano map shows a comparison of the content and *p* value of gene expression between NP and PE placental villus explants proteins. (E) The heatmap displays expression patterns of the top 10 differentially expressed proteins in NP and PE placental villus explants, stratified by molecular weight (<30, 30–50, and >50 kDa). (F) Western blot was used to determine the expression levels of HSP27 and HRS in 293T cells. (G) 293T cells, either wild‐type or HSP27‐knockdown, were co‐transfected with an HA‐Ub expression plasmid. Following MG132 treatment, cells were harvested, and the expression levels of HRS along with its ubiquitination status were analyzed.

Although the protein level of HRS in placental villi from PE patients was significantly lower than that from NP patients (Figure 6C,D), its RNA level showed no significant change (Figure S10I), suggesting that the reduction in HRS may result from increased degradation. To explore the mechanism underlying HRS degradation, placental villus lysates from NP and PE patients were added to cultured cells. We found that lysates from PE patients significantly reduced HRS expression levels in placental explants and HTR‐8/SVNEO cells (Figure 8A). Further investigation revealed that proteins with a molecular weight below 30 kDa in PE placental villus lysates were responsible for the decreased HRS levels (Figure 8B). We performed protein mass spectrometry analysis on NP and PE placental villi proteins and identified significant differences between the two groups (Figure 8C). A total of 159 differentially expressed proteins (DEPs) were identified in PE placental lysates, with 47 upregulated and 112 downregulated (Figure 8D). Among proteins with a molecular weight below 30 kDa, HSP27 expression was significantly higher in PE patients compared to NP patients (Figure 8E).

Hypoxia or oxidative stress in tissues often leads to a significant increase in heat shock protein levels [35, 36]. Ischemia/reperfusion injury and oxidative stress, which are key pathological features of the placenta in PE patients, may be critical factors driving the upregulation of HSP27 expression [37, 38]. We observed that HSP27 overexpression in cells significantly reduced intracellular HRS levels, and this effect was reversed by the proteasome inhibitor MG132 but not by the lysosome inhibitor chloroquine (CQ) (Figure 8F). Given that the ubiquitin‐proteasome pathway represents the major protein degradation mechanism and considering the established role of HSP27 in mediating target protein ubiquitination [39, 40], we subsequently explored this regulatory pathway. Knockdown of HSP27 in cells increased intracellular HRS levels and reduced its ubiquitination, and this effect was reversed by MG132 (Figure 8G).

These findings demonstrate that elevated HSP27 expression in trophoblasts from PE placental trophoblasts induces ubiquitination‐dependent degradation of HRS.

## 3 Discussion

PE is a placental disease characterized by complex underlying mechanisms [41]. Among these, alterations in pEVs have become a pivotal research focus for delving into PE pathogenesis [3]. Nevertheless, the molecular mechanisms underlying these vesicular changes and their precise pathophysiological contributions to PE development remain incompletely characterized. This study revealed that decreased NAM levels in pEVs disrupt the maternal‐fetal immune microenvironment by increasing frequencies of Th1 and Th17 cells, thereby driving PE progression. Moreover, the decrease of NAM in pEVs results from enhanced HRS degradation, which is mediated by upregulated HSP27 expression in placental trophoblasts.

Placental EVs (pEVs), mainly constituted by PLAP^+^ EVs, contain diverse bioactive cargo including proteins, RNAs, and metabolites [42, 43] Through experiments involving protein or RNA depletion of NP‐EVs followed by co‐culture with CD4^+^ T cells, we demonstrated that the immunomodulatory capacity of NP‐EVs persists even after RNA and protein degradation. Through metabolomics analysis of NP‐EVs and PE‐EVs, along with adenoviral shRNA knockdown and PA‐mediated inhibition to decrease NAM levels in pEVs, we demonstrated that pEV‐associated NAM plays an important role in immune regulation.

To demonstrate that the impaired ability of PE‐EVs to induce maternal‐fetal immune tolerance is due to the decline in NAM levels, we engineered EV‐mimetic liposomes encapsulating NAM (Lipo‐NAM) and blank control liposomes (Lipo‐blank). Functional characterization revealed that Lipo‐NAM significantly attenuated inflammatory responses in CD4^+^ T cells compared with Lipo‐blank. In vivo studies utilizing adenoviral shRNA knockdown and PA‐mediated inhibition mice models demonstrated that pEV NAM deficiency disrupts CD4^+^ T cells immune homeostasis at the maternal‐fetal interface, thereby contributing to PE pathogenesis. To pinpoint the critical CD4^+^ T cell subset mediating pEVs' effects, we performed in vivo cytokine neutralization targeting IFN‐γ (primarily produced by Th1 cells) and IL‐17A (mainly secreted by Th17 cells). The more pronounced alleviation of PE‐like symptoms with IFN‐γ blockade implicates Th1 cells as the dominant contributors to pEVs‐induced pathogenesis.

Beyond T cells and macrophages (the predominant immune populations in the placenta), natural killer (NK) cells and dendritic cells (DCs) also contribute to the placental immune microenvironment. Emerging evidence indicates that NAM exerts cell type‐ and tissue‐specific immunomodulatory effects. While NAM enhances the cytotoxic activity of peripheral NK cells in oncology contexts [44], decidual NK (dNK) cells serve distinct roles in pregnancy, primarily secreting trophic factors to support fetal development [45]. We hypothesize that NAM may preferentially augment dNK cells’ secretory function over cytotoxicity, thereby favoring pregnancy maintenance. Similarly, DCs overactivation can impair maternal‐fetal tolerance [46]. Clinical evidence shows NAM suppresses DC‐driven inflammation in psoriasis [47], suggesting it may likewise modulate DC activity at the maternal‐fetal interface to sustain immune equilibrium and prevent pathological inflammation.

In addition, insufficient trophoblast invasion is a major contributor to the development of PE [11]. Although knockdown of Rab27a (inhibiting EV secretion) or HRS (reducing NAM levels in EVs) in trophoblasts did not directly impair their invasive capacity in vitro, we observed in vivo that reduced NAM levels in pEVs led to a decrease tendency in the labyrinth zone (LZ) to trophoblast zone (TZ) ratio in mouse placentas. Furthermore, in vitro experiments demonstrated that the supernatant from CD4^+^ T cells treated with low‐NAM pEVs significantly suppressed trophoblast invasion compared to the supernatant from control group CD4^+^ T cells. These findings suggest that pEVs may also impaired trophoblast invasion by modulating immune cell cytokine secretion, thereby promoting PE. These results improved understanding of NAM's role, and provides new mechanistic insights into its potential therapeutic value for pregnancy complications.

The tissue‐specific origin and systemic distribution of EVs render them particularly suitable for liquid biopsy‐based diagnostic approaches [48]. And PE poses significant clinical challenges due to the absence of reliable predictive biomarkers [2]. While NAM supplementation improves pregnancy outcomes in preclinical models [15, 49], its oral bioavailability and hepatic metabolism limit therapeutic efficacy at the maternal‐fetal interface [50]. Notably, we found NAM enrichment in pEVs compared to placental tissue and maternal serum, suggesting their potential as both diagnostic markers and targeted delivery vehicles. Building on evidence that placental chondroitin sulfate A‐binding peptide (plCSA‐BP) selectively targets trophoblasts [51], future efforts should develop plCSA‐BP‐or similar targeting ligands‐modified, NAM‐loaded EVs for placental delivery while evaluating combined PLAP^+^ EV NAM levels with existing biomarkers for early PE detection.

Although reduced NAM levels in pEVs have been clearly implicated in PE progression, the mechanisms driving this decrease remain unclear. While the ESCRT machinery is known to mediate protein sorting into EVs [52], the loading of metabolic cargo (such as NAM) into trophoblast multivesicular bodies (MVBs) and its subsequent incorporation into pEVs remains poorly characterized. In our study, we reveal that NAM selectively binds to the ESCRT‐0 component HRS. Notably, among multiple ESCRT‐associated proteins (e.g., TSG101, Alix), only HRS expression was dysregulated in placental villi from PE patients compared with those from NP. Further experiments demonstrated that depletion of HRS in trophoblasts not only reduced the level of NAM in secreted extracellular vesicles (EVs) but also altered the morphology of intracellular multivesicular bodies (MVBs). Subsequent investigations confirmed that NAM binds directly to the tryptophan 115 (W115) residue of HRS. We observed that mutation of W115 to alanine, which abolishes its function, significantly impaired the binding between NAM and HRS. Taken together, these findings establish HRS as a key regulator responsible for NAM loading into placental EVs (pEVs).

HRS protein levels were decreased in PE placentas despite unaltered mRNA expression, implying the reduction may be due to increased protein degradation. Using ultrafiltration to isolate proteins from placental villus explants by molecular weight, we demonstrated that proteins below 30 kDa from PE patients reduced HRS levels in trophoblast cells. By combining mass spectrometry analysis of low‐molecular‐weight (<30 kDa) differentially expressed proteins in NP and PE placental villi with immunoprecipitation of HRS ubiquitination levels in HSP27‐modulated trophoblasts, we identified HSP27—a member of the small heat shock protein family [38, 39]—as significantly upregulated in PE placental villi. Further mechanistic studies revealed that HSP27 promotes HRS degradation via the proteasome, as evidenced by rescue with the proteasome inhibitor MG132 but not with the lysosome inhibitor chloroquine (CQ). The ubiquitin‐proteasome pathway is the major mechanism of protein degradation. [53] Since hypoxia and oxidative stress are established inducers of heat shock proteins, [35, 36] the PE placental microenvironment likely drives HSP27 overexpression [54], ultimately leading to reduced HRS stability. Together, these findings elucidate the mechanism underlying decreased HRS levels in PE trophoblasts.

Our study demonstrates that NAM is enriched in pEVs and plays a critical role in maintaining maternal‐fetal immune tolerance. Mechanistically, NAM in pEVs inhibits Th1 differentiation via SIRT1 suppression and Th17 differentiation via macrophages. We further identify that diminished NAM levels in PE‐EVs result from HSP27‐mediated HRS degradation in trophoblasts. These findings offer novel insights into the pathogenesis of PE and suggest potential therapeutic avenues for its intervention.

## 4 Materials and Methods

### 4.1 Clinical Sample Collection

The samples used in this study were collected from Sir Run Run Shaw Hospital, Zhejiang University School of Medicine, between October 2020 and August 2024. Human placentas were obtained from women with NP or PE who underwent elective cesarean delivery. PE was defined as new‐onset hypertension (systolic blood pressure ≥140 mmHg and/or diastolic blood pressure ≥90 mmHg) after 20 weeks of gestation, accompanied by proteinuria (≥300 mg on at least two occasions). All PE cases in this study were late‐onset (defined as occurring after 34 weeks of gestation) [55].

Control subjects consisted of women with normal blood pressure, full‐term pregnancies, and no pregnancy‐related complications. The detailed clinical characteristics of the pregnant women in this study are presented in Table 1.

**TABLE 1:** Clinical characteristics of the NP and PE patients included in the study.

Parameters	Normal pregnancy (*n* = 40)	Pre‐eclampsia (*n* = 33)	*p* Value
Age (years)	31.95 ± 3.22	32.30 ± 4.33	0.6826
BMI (kg/m^2^)	26.75 ± 2.10	28.45 ± 3.67	0.0132
Gestational age (weeks)	38.96 ± 0.58	36.75 ± 1.72	<0.0001
Number of living children	1.70 ± 0.52	1.50 ± 0.64	0.1282
Systolic blood pressure	111.2 ± 10.00	147.4 ± 11.59	<0.0001
Diastolic blood pressure	73.64 ± 7.44	94.15 ± 6.44	<0.0001

All clinical samples used in this study were collected with patients' informed consent, including consent for publication. This study complied with: Regulations on Ethical Review of Biomedical Research Involving Humans (2016, National Health Commission of China); WMA Declaration of Helsinki; CIOMS International Ethical Guidelines for Biomedical Research. The study protocol was approved by the Medical Ethics Committee of Sir Run Run Shaw Hospital, Zhejiang University (Approval No: 20220207‐30).

### 4.2 Laboratory Mice

Eight‐week‐old female C57BL/6 mice and ten‐week‐old male BALB/c mice were obtained from Hangzhou Ziyuan Laboratory Animal Technology Co., Ltd. (Hangzhou, China) and Shanghai Jihui Experimental Animal Breeding Co., Ltd. (Shanghai, China), respectively. All animals were maintained under specific pathogen‐free conditions. An allogeneic pregnancy model was established by mating female C57BL/6 mice with male BALB/c mice [56]. Gestational day 0.5 (GD0.5) was designated upon vaginal plug detection. Systolic blood pressure (SBP) was measured at GD12.5 and GD16.5 using a noninvasive tail‐cuff system (BP‐2010A, Softron, Japan). Three random urine samples were collected after GD16.5 for urinary albumin‐to‐creatinine ratio (UACR) determination using a commercial assay kit (CH0101060, Maccura, China). All mice were euthanized at GD 18.5.

All experimental procedures were performed in accordance with the Chinese Guidelines for the Care and Use of Laboratory Animals and were approved by the Animal Research Ethics Committee of Sir Run Run Shaw Hospital, Zhejiang University (Approval No: SRRSH202402608).

### 4.3 Cell Lines

The human trophoblast cell line HTR8/SVneo (RRID:CVCL_7162) were commercially acquired from the American Type Culture Collection (ATCC, USA), while the choriocarcinoma cell line JEG3 (RRID:CVCL_0363) were obtained from the Chinese Academy of Sciences. HTR8 cells were grown in RPMI‐1640 basal medium (Gibco, USA) containing 10% fetal bovine serum (FBS; Gibco, USA) and 1% penicillin–streptomycin (Gibco, USA; Cat# 15140122). JEG3 cells were cultured under similar conditions using DMEM/F12 medium (Gibco, USA) with identical serum and antibiotic supplements.

HEK293T human embryonic kidney cells (RRID:CVCL_0063) were obtained from the Chinese Academy of Sciences and maintained in DMEM (Gibco, USA; Cat# C11995500BT) supplemented with 10% FBS (CellMax, China; Cat# SA101.01) and 1% penicillin‐streptomycin.

All cell lines were incubated at 37 °C in a 5% CO_2_ humidified incubator. Mycoplasma contamination tests were performed monthly to ensure cell lines were contamination free. Culture media were refreshed every 48 h, and cells were subcultured at 70–80% confluence to maintain optimal growth conditions.

### 4.4 An Animal Model of Reducing Uterine Perfusion Pressure (RUPP)

The detailed methodology for establishing the reduced uterine perfusion pressure (RUPP) PE mouse model has been described in previous publication [57]. And in our 2025 *eLife* study, we demonstrated that the RUPP model effectively recapitulates PE [21]. Briefly, we performed unilateral uterine artery ligation on gestational day (GD) 12.5 to establish a preeclampsia (PE) mouse model. Pregnant mice were anesthetized via intraperitoneal injection of 4% chloral hydrate. Following bilateral dorsal incisions, surgical sutures were placed to partially restrict blood flow in the uterine arcades.

### 4.5 An Animal Model of Intrauterine Administration of Rab27a/QPRT Adenovirus or GW4869 or Phthalic Acid

Uterine Cavity Perfusion Protocol: Firstly, anesthetize mice via intraperitoneal injection of Avertin (200 mg/kg). Then, perform uterine perfusion using a gavage needle stainless steel inserted through the vagina. Post‐infusion, position the mouse at a 30° head‐down tilt (hind limbs elevated) for 30 min to ensure proper retention.

On gestational days (GD) 10.5 and 14.5, pregnant rats received intrauterine administration of: 50 µL of adenovirus encoding either Rab27A or *QPRT* or Negative control (10^11^ copies/mL); GW4869 (1 mg/kg, S7609, Selleck, USA) or DMSO (vehicle control); Phthalic acid (P815673, MACKLIN, China) or 5% ethanol (vehicle control).

On GD 11.5 and 15.5, mice were intraperitoneally injected with either 200 µg/200 µL pEVs or PBS (control). All animals were euthanized at GD 18.5.

### 4.6 An Animal Model of Exogenous Supplement of Nicotinamide (NAM)

For the NAM supplementation group, mice received intraperitoneal injections of PE‐EVs mixed with NAM (500 mg/kg; N814697, MACKLIN, USA). The NAM concentration was selected based on established literature reports [15].

### 4.7 An Animal Model of Tail Vein Infusion of CD4^+^ T Cells

On GD 10.5 and 14.5, endogenous CD4+ T cells were depleted through intraperitoneal injection of anti‐CD4 antibody (200 µg per mouse, BE0003‐1, BioXcell, USA).

For adoptive transfer experiments, CD4^+^ T cells were isolated from spleens of non‐pregnant C57BL/6 mice using a Mouse CD4^+^ T Cell Isolation Kit (#19852; STEMCELL Technologies). Then, Isolated cells were pretreated for 3 days with either 100 × 10^−9^ m SRT1720 (HY‐10532; MedChemExpress, USA), or DMSO. Following pretreatment, cells were coincubated with NP‐EVs for 3 days. Treated CD4^+^ T cells (5×10⁶) were then adoptively transferred into pregnant mice via intravenous injection 24 h after anti‐CD4 antibody administration.

### 4.8 An Animal Model of Tail Vein Infusion of pEVs Treated Bone Marrow‐Derived Macrophages (BMDMs)

Bone marrow‐derived macrophages (BMDMs) were isolated and cultured as previously described [58]. These BMDMs were then co‐cultured for 24 h with pEVs (100 µg/mL) containing different concentrations of NAM. For in vivo experiments, on GD 10.5 and 14.5 pregnant mice received intraperitoneal injections of either 200 µL clodronate liposomes (40337ES10; YEASEN; to deplete endogenous macrophages), or clodronate control (40338ES10; YEASEN, China). On GD 11.5 and 15.5, mice were intravenous injected with 1×10⁶ BMDMs (in 200 µL PBS) via tail vein.

### 4.9 Isolation of Human Placental Lymphocytes and pEVs

Placental villi rather than chorionic plate and extraplacental membranes were used in this part of the study. Placentas were washed twice with saline and cut into small pieces with aseptic scissors. Then, we placed the cut pieces of tissue into 50 mL centrifuge tubes. And appropriate amounts of RPMI 1640 medium (Thermo Fisher Scientific), type IV collagenase (1 mg mL, Sigma‐Aldrich, USA) and DNase I (0.01 mg mL, Sigma–Aldrich, USA) were added. Centrifuge tubes were fixed on a 37 °C shaker at 200 rpm and digested for 60 min. After digestion was completed, the tissue solution was filtered through a 70 µm nylon mesh to obtain a single cell suspension.

We obtained placenta‐derived EVs by centrifuging a single cell suspension at 10 000 g for 60 min, collecting the supernatant and filtering it using a 0.22 µm filter, then centrifuging it at 100 000 g for 1 h at 4°C and washing it once with PBS. Then, phycoerythrin‐conjugated PLAP (sc47691, Santa Cruz Biotechnology, USA) and Releasable Phycoerythrin Positive Kit (17865, Stem Cell, Canada) were used to obtain purified PLAP‐positive pEVs according to the manufacturer's instructions [8].

### 4.10 Degradation of NP‐EV Proteins and RNA by Proteases and RNases

NP‐EVs were treated with Trypsin/EDTA Solution (R001100, Thermo Fisher, USA) or RNase A (R1253, Thermo Fisher, USA) at 37°C for 5 min to degrade protein and RNA components. Subsequently, CD4^+^ T cells were cocultured with the processed NP‐EVs (100 µg) for 3 days for functional assays.

### 4.11 Preparation and Characterization of Liposomes

NAM loaded Liposomes were prepared using a film‐ultrasonic dispersion method [59]. Briefly, cholesterol, lecithin and 1,2‐distearoyl‐sn‐glycero‐3‐phosphoethanolamine‐*N*‐[methoxy(polyethylene glycol)‐2000] (DSPE‐mPEG), at a molar ratio of 10:20:1, was mixed and dissolved in 10 mL of dichloromethane and dried at the bottom of a flask as a thin film with a vacuum rotary evaporator under reduced pressure. Then, 10 mL of double‐distilled water consisting NAM was added to the flask. The hydrated the lipid was sonicated for 5 min using a sonicator. Unloaded drugs were removed by ultrafiltration device (COMW: 30 kDa). To avoid potential decomposition, the stock and working solutions NAM and Lipo‐NAM were freshly prepared and stored at 4°C less than a week.

### 4.12 Measurement of NAM Concentration in pEVs

pEVs were subjected to five freeze‐thaw cycles alternating between −80°C and 37°C to ensure complete lysis. The protein concentration of lysed pEVs was quantified using a BCA Protein Assay Kit II (23225; Thermo Fisher Scientific, USA), and samples were normalized to equal concentrations with PBS. NAM levels in mouse‐ or human‐derived pEVs were measured using a Mouse NAM ELISA Kit (mlbio, China) or Human NAM ELISA Kit (mlbio, China), respectively, following the manufacturers' protocols. The detailed clinical characteristics of the pregnant women providing pEVs for NAM quantification by ELISA are presented in Table 2.

**TABLE 2:** Clinical characteristics of NP and PE patients providing pEVs for NAM quantification by ELISA.

Parameters	Normal pregnancy (*n* = 15)	Pre‐eclampsia (*n* = 15)	*p* value
Age (years)	32.00 ± 4.17	32.07 ± 4.73	0.9676
BMI (kg/m^2^)	26.66 ± 2.76	27.97 ±1.96	0.1454
Gestational age (weeks)	38.70 ± 0.50	38.16 ± 0.93	0.0572
Number of living children	1.80 ± 0.42	1.80 ± 0.68	>0.9999
Systolic blood pressure	115.8 ± 9.56	151.63 ± 12.04	<0.0001
Diastolic blood pressure	75.20 ± 9.55	95.13 ± 7.23	0.0002

### 4.13 Untargeted Metabolomics of pEVs by LC–MS

Placental villi rather than chorionic plate and extraplacental membranes were used in this part of the study. An untargeted metabolomics analysis was performed on PLAP‐positive pEVs isolated from the placentas of normotensive pregnant (NP, *n* = 8) and preeclamptic (PE, *n* = 8) patients, who were rigorously matched for age, gestational age, and body mass index (clinical characteristics detailed in Table 3). Metabolomic profiling was conducted using liquid chromatography‐tandem mass spectrometry (LC‐MS/MS) at Shenzhen Huada Gene Technology Service Co., Ltd. (Shenzhen, China). Data acquisition was performed on a high‐resolution q Exactive mass spectrometer (Thermo Fisher Scientific) in both positive and negative ionization modes. Metabolites with a false discovery rate (FDR)‐corrected *p*‐value < 0.05 were considered statistically significant.

**TABLE 3:** Clinical characteristics of NP and PE patients providing pEVs for untargeted metabolomics.

Parameters	Normal pregnancy (*n* = 8)	Pre‐eclampsia (*n* = 8)	*p* value
age (years)	33.00 ± 3.67	34.00 ± 4.24	0.6217
BMI (kg/m^2^)	27.31 ± 1.99	27.75 ±4.27	0.7957
Gestational age (weeks)	38.89 ± 0.56	36.19 ± 1.42	0.0002
Number of living children	1.50 ± 0.53	1.63 ± 0.52	0.6420
Systolic blood pressure	111.7 ± 7.61	149.9 ± 15.67	<0.0001
Diastolic blood pressure	74.43 ± 8.83	95.14 ± 7.99	0.0006

### 4.14 RNA Transcriptome of CD4^+^ T Cells

Peripheral blood (10 mL) was collected from healthy non‐pregnant female volunteers. Lymphocytes were isolated by density gradient centrifugation using Ficoll‐Paque (P4350; Solarbio) and washed twice with PBS. After incubation in a 12‐well plate for 2 h at 37 °C, nonadherent cells were collected. CD4^+^ T cells were then positively selected using a Human CD4+ T Cell Isolation Kit II (17852; STEMCELL Technologies).

RNA sequencing was performed on CD4+ T cells treated for 3 days with either normotensive pregnancy‐derived EVs (NP‐EVs, *n* = 10) or preeclampsia‐derived EVs (PE‐EVs, *n* = 10). Library preparation and sequencing were conducted by Shenzhen Huada Gene Technology Service Co., Ltd. (Shenzhen, China) using Illumina platforms. Differentially expressed genes were identified based on the following criteria: fold change >2 and false discovery rate (FDR)‐adjusted *p*‐value <0.05.

### 4.15 Transmission Electron Microscopy (TEM)

pEVs (5‐10 µL) were dropped onto copper mesh and adsorbed for 10 min at room temperature. Then, absorbent paper was used to absorb the excess liquid. 10 µL of 2% uranium acetate was added to the copper mesh for 2 min in order to negative staining. Then, the cells were observed by transmission electron microscopy.

Following the well‐established protocol in the literature, trophoblast cells subjected to lentiviral‐mediated knockdown of HRS were imaged using TEM, and the morphological alterations within multivesicular bodies (MVBs) were observed.

### 4.16 Nanoparticle Tracking Analysis (NTA)

Extracellular vesicles diluted with PBS to the appropriate concentration and used NanoSight NS300 (Malvern, USA) to determine the size and diameter of pEVs at room temperature. The ZetaView system was used for data analysis. Polystyrene pellets were used to calibrate the ZetaView system.

### 4.17 Flow Cytometry Analyses of pEVs and Cells

First, we mix 20 µg of pEVs with 5 µL of 4‐µm aldehyde/sulfate latex beads (A37306, Invitrogen, U.S.A.) in a 1.5 mL EP tube. Incubate at room temperature for 15 min before adding 1 mL of PBS. Fix the EP tubes on a 4 °C shaker and incubate with shaking for 1 h before centrifuging at 3500 g for 5 min. The precipitate was blocked by incubation with 200 µL of fetal bovine serum for 30 min. Then incubated the beads with phycoerythrin‐conjugated anti‐human PLAP or phycoerythrin‐conjugated anti‐mouse CK7 for 30 min in the dark at room temperature. The beads were analyzed by FCM (Beckman Coulter).

For FCM analyses of cells, the single‐cell suspension was washed twice with PBS and stained with surface‐antibody at room temperature for 30 min. Then, after treatment with IC fixation Buffer (00‐8222‐49, Invitrogen, USA), the cells were stained with intracellular‐antibody at room temperature for 30 min. Finally, the cells were stained with secondary‐antibody at room temperature for 30 min. Finally, 1 mL of PBS was added and washed twice, and the precipitate was mixed with 200 µL PBS for flow analysis.

### 4.18 Western Blot

A total of 30 µg of pEVs were immunoblotted with the indicated antibodies. We separated the mixed protein samples by polyacrylamide gel electrophoresis (PAGE). Then we transfer the proteins onto polyvinylidene difluoride (PVDF) membranes (Millipore Corp., Billerica, MA, USA) afterwards. The proteins on PVDF are then used as antigens and specifically bound to their corresponding primary antibodies. Finally, the membranes were incubated with anti‐rabbit (7074, Cell Signaling Technology, USA) or anti‐mouse secondary antibodies (7076, Cell Signaling Technology, USA) and analyzed by enhanced chemiluminescence (WBKLS0500, Millipore Corp, Billerica, MA, USA) for visualization.

### 4.19 Measurement of NAD+/NADH Ratio in Macrophages

The level of NAD+/NADH ratio in macrophages treated with 100 µg Ad Sh‐NC‐EVs or Ad Sh‐*QPRT*‐EVs was measured using an NAD+/NADH Assay Kit with WST‐8 (S0175, Beyotime, China).

### 4.20 Visualization of the Interaction Pattern of NAM With SIRT1 and NAM with HRS

To evaluate the interaction pattern of NAM with SIRT1 and NAM with HRS, we first obtained the molecular structure of NAM from the pubchem compound database (http://pubchem.ncbi.nlm.nih.gov/) and then downloaded the SIRT1 protein and HRS protein structure from the PDB (http://www.rcsb.org/). Finally the interaction model was visualized using AutodockVina 1.2.2, a protein‐ligand docking software.

### 4.21 The Oxygen Consumption Rate (OCR) and Extracellular Acidification Rate (ECAR) Measurement

To quantify OCR and ECAR in macrophages, freshly procured BMDMs, at a density of 200,000 cells per well, were incubated with either Ad sh‐NC‐EVs or Ad sh‐*QPRT*‐EVs. Subsequently, the cells were seeded onto polylysine‐coated Seahorse plates immersed in XF medium, which comprised 25 × 10^−3^ M glucose, 2 × 10^−3^ M glutamine, and 1 × 10^−3^ pyruvate. Utilizing an XF‐8 Extracellular Flux Analyzer from Agilent Technologies (Santa Clara, California, USA), the metabolic profiles of these cells were analyzed.

For OCR assessment, the cells were sequentially exposed to 2 × 10^−3^ ‐ oligomycin, 1.5 × 10^−3^ ‐m FCCP, 1 × 10^−3^ ‐m rotenone, and 1 × 10^−6^ m antimycin A (Agilent Technologies). To determine ECAR, the cells were subjected to additions of glucose (10 × 10^−3^ ‐m), oligomycin A (1 × 10^−3^ ‐m), and 2‐DG (50 × 10^−3^ m).

### 4.22 Transfection with Lentivirus

Seed cells at a density of 100 000 cells per well of 24‐well plate at the time before transfection. The following day, the next day, the original medium was replaced with 2 mL of fresh medium containing 6 µg mL^−1^ polybrene, and an appropriate amount of viral suspension was added at an MOI of 50. After 24 h of incubation at 37 °C, replace the virus‐containing medium with fresh medium. To select for successfully transduced cells, supplement the fresh medium with 2 µg mL^−1^ of puromycin and maintain the cells under this selective condition for three consecutive passages, thereby generating stably transduced cell lines.

### 4.23 Immunoprecipitation

**SCHEME 1:**
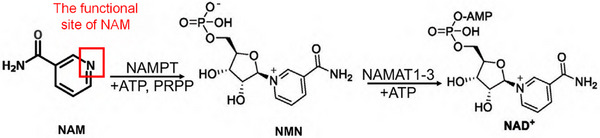
Schematic diagram of the intracellular conversion pathway of NAM to NAD+, with the functional group of NAM highlighted in the red box.

**SCHEME 2:**
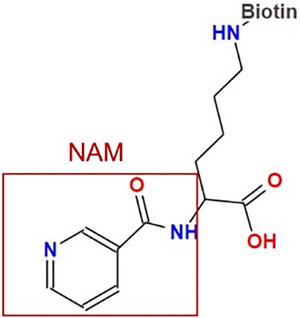
Structural schematic illustrating the conjugation of NAM and Biotin via a linker arm.

The functional site of NAM (indicated by the red box in the Scheme 1) can be converted into NAD+ in vivo. Biotin was conjugated to the non‐functional site of NAM (as shown in Scheme 2) to obtain biotin‐conjugated NAM. Biotin‐conjugated NAM or IgG‐conjugated NAM (100 µg) was added into either villus explants (100 mg) or HEK293T cells (12‐well plate at a density of 1 × 10⁶ cells per well), and subsequently, the cells were lysed on ice after a 2‐h incubation. Following this, magnetic beads that could bind biotin were added, enabling the capture and adsorption of NAM‐bound proteins onto a magnetic stand. The methodology for these steps has been documented in the relevant literature [60].

HEK293T cells were plated in a 12‐well plate at a density of 1 × 10⁶ cells per well (in 1 mL complete medium) and allowed to adhere overnight. Then, transfection complex were prepared. Tube A: Opti‐MEM (50 µL), 50 × 10^−9^ m siRNA (targeting HSPB1 or nontargeting control, NC‐siRNA) and 1 µg/well HA‐tagged ubiquitin (HA‐Ub) plasmid; Tube B: Opti‐MEM (50 µL), Lipofectamine 3000 (2 µL/µg DNA). Tubes A and B were mixed gently and incubated at room temperature for 15 min to form transfection complexes.

The original culture medium was aspirated, and cells were washed once with PBS. Serum‐free medium (500 µL) was added to each well. The transfection complexes (100 µL total) were added dropwise to the wells and gently mixed. After 12 h, the medium was replaced with complete medium to reduce cytotoxicity. At 48 h post‐transfection, cells were treated with 10 × 10^−6^ m MG132 or chloroquine (CQ) for 6 h to accumulate ubiquitinated proteins. Cells were lysed, and total protein was extracted for Western blot analysis. Ubiquitination levels were detected using an anti‐HA antibody (to visualize HA‐Ub‐conjugated proteins). HRS levels were assessed in HEK293T cells after MG132 treatment.

### 4.24 Confocal Microscopy

**SCHEME 3:**
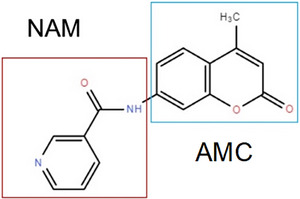
Structural schematic illustrating the conjugation of NAM and AMC.

Fluorescent group 7‐amino‐4‐methylcoumarin (AMC) was conjugated to the nonfunctional site of NAM (as shown in Scheme 3) to obtain AMC‐conjugated NAM. HEK293T cells were plated in a 12‐well plate at a density of 1 × 10⁶ cells per well (in 1 mL complete medium). Then AMC‐conjugated NAM (100 µg) was added into 293T cells for 2 h.

Following three gentle PBS washes, cells were fixed with 4% paraformaldehyde (PFA) at room temperature for 15 min, then washed again. Permeabilization was performed using 0.3% Triton X‐100 (in PBS) for 20 min at room temperature. To reduce nonspecific binding, cells were blocked with 5% BSA (or goat serum) for 1 h at room temperature. Cells were then incubated with primary antibody against HRS (1:100; sc‐271455; Santa Cruz Biotechnology) followed by secondary antibody (anti‐mouse 549; GAM492; MULTI SCIENCES). Nuclei were counterstained with DAPI, and images were acquired using a Zeiss LSM800 confocal laser microscope.

### 4.25 Molecular Weight Fractionation of Placental Villus Proteins Using Ultrafiltration

Placental villi rather than chorionic plate and extraplacental membranes were used in this part of the study. Weigh 1 g of placental villi (from NP or PE patients) and add 5 mL ice‐cold PBS (pH 7.4) and sterile zirconium grinding beads (0.5 mm diameter). Homogenize placental villi using a prechilled tissue grinder at 4 °C until no visible tissue fragments, then centrifuge at 3000 × g for 30 min at 4 °C and collect supernatant as crude placental villus protein lysate. Prepare centrifugal filter units (Amicon Ultra, USA) with: 30 kDa MWCO, 50 kDa MWCO, 100 kDa MWCO. Load 500 µL of lysate onto each filter and centrifuge at 1500 × g for 10 min at 4 °C. The retentate (>MWCO) is protein solution above membrane, whike the filtrate (< MWCO) is flow‐through below membrane.

### 4.26 Protein Mass Spectrometry of Placental Villus Explant

Placental villi rather than chorionic plate and extraplacental membranes were used in this part of the study. Mass spectrometry was conducted on the proteins within normal control villus explants that bind with Biotin‐conjugated NAM or IgG‐conjugated NAM at Shenzhen Huada Gene Technology Service Co. Ltd. (Shenzhen, China).

Furthermore, a mass spectrometry investigation was carried out on the placental lysate of NP (*n* = 6) and PE (*n* = 6) patients, conducted by Shenzhen Huada Gene Technology Service Co. Ltd. (Shenzhen, China). The detailed clinical characteristics of the pregnant women are presented in Table 4.

**TABLE 4:** Clinical characteristics of NP and PE patients providing placental villi for Protein mass spectrometry.

Parameters	Normal pregnancy (*n* = 6)	Pre‐eclampsia (*n* = 6)	*p* Value
Age (years)	32.17 ± 2.71	32.83 ± 4.31	0.7551
BMI (kg/m^2^)	26.92 ± 1.94	26.59 ± 4.36	0.8679
Gestational age (weeks)	38.80 ± 0.64	36.31 ± 1.48	0.0035
Number of living children	1.50 ± 0.55	1.33 ± 0.52	>0.9999
Systolic blood pressure	111.6 ± 8.26	154.4± 16.63	0.0010
Diastolic blood pressure	75.00 ± 9.43	97.60 ± 8.29	0.0055

### 4.27 Assessment of Cell Invasive Capacity Using a Matrigel‐Coated Transwell Assay

We used a Transwell‐Matrigel assay to test cell invasion. The inserts were coated with Matrigel (BD Biosciences) to mimic a basement membrane. Cells were placed in the upper chamber in a serum‐free medium, while a medium with serum was added below to attract them. After 24 h, we removed the non‐invading cells from the top. The cells that had invaded through the Matrigel to the bottom side were stained and counted under a microscope.

### 4.28 Statistical Analysis

All statistical analyses were performed using SPSS v20.0 (IBM, USA) and GraphPad Prism v6.0 (GraphPad, USA). Data normality was first assessed using the Shapiro‐Wilk test. For normally distributed data, comparisons between two groups were conducted using Student's *t*‐test, while multiple group comparisons were performed using one‐way ANOVA followed by post hoc tests. For non‐normally distributed data, the Kruskal–Wallis test were used for multiple groups comparisons. All data are presented as mean ± standard error of the mean (SEM), with statistical significance denoted as *p < 0.05, **p < 0.01, ***p < 0.001, and ****p < 0.0001; NS indicates not statistically significant  .

## Author Contributions

H.F., Y.L., and L.J. contributed equally to this work and share first authorship. H.F. and L.J. conceived and designed the study. X.L., Z.S., and X.L. established the animal models. X.Z. collected the clinical samples. H.F., Y.L., X.L., X.L., and F.J. performed the experiments and analyzed the data. H.F. and Z.S. interpreted the results. Y.L. and S.X. wrote the manuscript draft. H.F. and Y.L. prepared the figures. L.J. and L.F. edited the manuscript. S.Z., L.J., H.F., and X.L. provided funding. S.Z. and L.J. cosupervised the work.

## Conflicts of Interest

The authors declare no conflicts of interest.

## Supporting information




**Supporting File**: advs73263‐sup‐0001‐SuppMat.docx.

## Data Availability

The data that support the findings of this study are available from the corresponding author upon reasonable request.

## References

[1] A. T. Dennis , A. Xin , and M. K. Farber , “Perioperative Management of Patients With Preeclampsia: A Comprehensive Review,” Anesthesiology 142, no. 2 (2025): 378–402. 39807917 10.1097/ALN.0000000000005296

[2] K. G. Tlaye , G. G. Woldeamanuel , K. C.‐Y. Wong , et al., “Pharmacogenomics and Pharmacokinetics of Aspirin in Preeclampsia Prevention,” Circulation Research 137, no. 1 (2025): 69–82. 40329906 10.1161/CIRCRESAHA.124.325699

[3] Z. Wang , G. Zhao , M. Zeng , W. Feng , and J. Liu , “Overview of Extracellular Vesicles in the Pathogenesis of Preeclampsia,” Biology of Reproduction 105, no. 1 (2021): 32–39. 33778844 10.1093/biolre/ioab060

[4] E. I. Buzas , “The Roles of Extracellular Vesicles in the Immune System,” Nature Reviews Immunology 23, no. 4 (2023): 236–250. 10.1038/s41577-022-00763-8 PMC9361922 35927511

[5] E. Deer , O. Herrock , N. Campbell , et al., “The Role of Immune Cells and Mediators in Preeclampsia,” Nature Reviews Nephrology 19, no. 4 (2023): 257–270. 36635411 10.1038/s41581-022-00670-0 PMC10038936

[6] C. Park , S. Alahari , J. Ausman , et al., “Placental Hypoxia‐Induced Ferroptosis Drives Vascular Damage in Preeclampsia,” Circulation Research 136, no. 4 (2025): 361–378. 39846172 10.1161/CIRCRESAHA.124.325119

[7] M. Gill , C. Motta‐Mejia , N. Kandzija , et al., “Placental Syncytiotrophoblast‐Derived Extracellular Vesicles Carry Active NEP (Neprilysin) and Are Increased in Preeclampsia,” Hypertension 73, no. 5 (2019): 1112–1119. 30929513 10.1161/HYPERTENSIONAHA.119.12707

[8] X. Liu , H. Fei , C. Yang , et al., “Trophoblast‐Derived Extracellular Vesicles Promote Preeclampsia by Regulating Macrophage Polarization,” Hypertension 79, no. 10 (2022): 2274–2287. 35993233 10.1161/HYPERTENSIONAHA.122.19244

[9] T. Awoyemi , C. Motta‐Mejia , W. Zhang , et al., “Syncytiotrophoblast Extracellular Vesicles from Late‐Onset Preeclampsia Placentae Suppress Pro‐Inflammatory Immune Response in THP‐1 Macrophages,” Frontiers in Immunology 12 (2021): 676056. 34163477 10.3389/fimmu.2021.676056 PMC8215361

[10] K. Bai , X. Li , Y. Guo , et al., “The Regulatory Role of Placental Extracellular Vesicle on Trophoblast and Endothelial Cell Functions,” Frontiers in Cell and Developmental Biology 13 (2025): 1528714. 39996028 10.3389/fcell.2025.1528714 PMC11847863

[11] M. Horvat Mercnik , C. Schliefsteiner , G. Sanchez‐Duffhues , et al., “TGFβ Signalling: A Nexus Between Inflammation, Placental Health and Preeclampsia throughout Pregnancy,” Human Reproduction Update 30, no. 4 (2024): 442–471. 38519450 10.1093/humupd/dmae007 PMC11215164

[12] M. Mittelbrunn , I. Nazarenko , E. N. Nolte‐'t Hoen , et al., “Biological Properties of Extracellular Vesicles and Their Physiological Functions,” Journal of Extracellular Vesicles 4 (2015): 27066. 25979354 10.3402/jev.v4.27066 PMC4433489

[13] F. M. Walocko , A. E. Eber , J. E. Keri , M. A. AL‐Harbi , and K. Nouri , “The Role of Nicotinamide in Acne Treatment,” Dermatologic Therapy 30, no. 5 (2017): 12481. 10.1111/dth.12481 28220628

[14] I. P. Nikas , S. A. Paschou , and H. S. Ryu , “The Role of Nicotinamide in Cancer Chemoprevention and Therapy,” Biomolecules 10, no. 3 (2020): 477. 32245130 10.3390/biom10030477 PMC7175378

[15] F. Li , T. Fushima , G. Oyanagi , et al., “Nicotinamide Benefits both Mothers and Pups in Two Contrasting Mouse Models of Preeclampsia,” Proceedings of the National Academy of Sciences 113, no. 47 (2016): 13450–13455. 10.1073/pnas.1614947113 PMC5127378 27821757

[16] R. Kalluri and V. S. LeBleu , “The Biology, Function, and Biomedical Applications of Exosomes,” Science 367, no. 6478 (2020): aau6977. 10.1126/science.aau6977 PMC7717626 32029601

[17] Y. Kuchitsu , K. Mukai , R. Uematsu , et al., “STING Signalling Is Terminated Through ESCRT‐dependent Microautophagy of Vesicles Originating From Recycling Endosomes,” Nature Cell Biology 25, no. 3 (2023): 453–466. 36918692 10.1038/s41556-023-01098-9 PMC10014584

[18] C. Tersigni , N. Di Simone , D. Lucchetti , et al., “Shedding of Syncytiotrophoblast‐Derived Extracellular Vesicles Is Increased in Placenta Previa and Accreta Spectrum,” Reproductive Sciences 31, no. 7 (2024): 2043–2048. 38453772 10.1007/s43032-024-01491-1 PMC11217103

[19] S. B. Noonan , S. P. Brennecke , and G. D. Jones , “Predicting Preeclampsia in Gestational Diabetes Mellitus Using the sFlt‐1/PlGF Ratio,” The Journal of Clinical Endocrinology & Metabolism 110 (2025): e3343–e3352. 39903607 10.1210/clinem/dgaf069

[20] G. Wang , J. Li , L. Bojmar , et al., “Tumour Extracellular Vesicles and Particles Induce Liver Metabolic Dysfunction,” Nature 618, no. 7964 (2023): 374–382. 37225988 10.1038/s41586-023-06114-4 PMC10330936

[21] H. Fei , X. Lu , Z. Shi , et al., “Deciphering the Preeclampsia‐specific Immune Microenvironment and the Role of Pro‐inflammatory Macrophages at the Maternal‐fetal Interface,” Elife 13 (2025): RP100002. 40152904 10.7554/eLife.100002 PMC11952753

[22] N. Berger , T. van der Wel , B. Hirschmugl , et al., “Inhibition of Diacylglycerol Lipase β Modulates Lipid and Endocannabinoid Levels in the Ex Vivo human Placenta,” Frontiers in Endocrinology 14 (2023): 1092024. 36864832 10.3389/fendo.2023.1092024 PMC9971001

[23] X. Ge , Q. Meng , L. Wei , et al., “Myocardial ischemia‐reperfusion induced cardiac extracellular vesicles harbour proinflammatory features and aggravate heart injury,” Journal of Extracellular Vesicles 10, no. 4 (2021): 12072. 10.1002/jev2.12072 PMC7902529 33664937

[24] A. J. Clark , M. C. Saade , V. Vemireddy , et al., “Hepatocyte Nuclear Factor 4α Mediated Quinolinate Phosphoribosylltransferase (QPRT) Expression in the Kidney Facilitates Resilience Against Acute Kidney Injury,” Kidney International 104, no. 6 (2023): 1150–1163. 37783445 10.1016/j.kint.2023.09.013 PMC10843022

[25] A. J. Covarrubias , R. Perrone , A. Grozio , and E. Verdin , “NAD+ Metabolism and Its Roles in Cellular Processes During Ageing,” Nature Reviews Molecular Cell Biology 22, no. 2 (2021): 119–141. 33353981 10.1038/s41580-020-00313-x PMC7963035

[26] S. S. Malik , D. N. Patterson , Z. Ncube , and E. A. Toth , “The Crystal Structure of Human Quinolinic Acid Phosphoribosyltransferase in Complex With Its Inhibitor Phthalic Acid,” Proteins: Structure, Function, and Bioinformatics 82, no. 3 (2014): 405–414. 10.1002/prot.24406 24038671

[27] Y. Wu , X. Wang , L. Song , et al., “Tuning Macrophage Phenotype for Enhancing Patency Rate and Tissue Regeneration of Vascular Grafts,” Acta Biomaterialia 198 (2025): 245–256. 40158766 10.1016/j.actbio.2025.03.053

[28] B. Li , C. Xia , W. He , et al., “The Thyroid Hormone Analog GC‐1 Mitigates Acute Lung Injury by Inhibiting M1 Macrophage Polarization,” Advanced Science 11, no. 44 (2024): 2401931. 39373388 10.1002/advs.202401931 PMC11600256

[29] Y. Zhang , J. Shi , J. Zhu , et al., “Immunometabolic Rewiring in Macrophages for Periodontitis Treatment via Nanoquercetin‐mediated Leverage of Glycolysis and OXPHOS,” Acta Pharmaceutica Sinica B 14, no. 11 (2024): 5026–5036. 39664434 10.1016/j.apsb.2024.07.008 PMC11628840

[30] Z. Qin , X. Fang , W. Sun , et al., “Deactylation by SIRT1 Enables Liquid–Liquid Phase Separation of IRF3/IRF7 in Innate Antiviral Immunity,” Nature Immunology 23, no. 8 (2022): 1193–1207. 35879450 10.1038/s41590-022-01269-0

[31] S. Pan , J. Leng , X. Deng , et al., “Nicotinamide Increases the Sensitivity of Chronic Myeloid Leukemia Cells to Doxorubicin via the Inhibition of SIRT1,” Journal of Cellular Biochemistry 121, no. 1 (2020): 574–586. 31407410 10.1002/jcb.29303

[32] C.‐C. Chao , C.‐L. Huang , J.‐J. Cheng , et al., “SRT1720 as an SIRT1 Activator for Alleviating Paraquat‐induced Models of Parkinson's Disease,” Redox Biology 58 (2022): 102534. 36379180 10.1016/j.redox.2022.102534 PMC9663539

[33] X. Ren and J. H. Hurley , “VHS Domains of ESCRT‐0 Cooperate in High‐Avidity Binding to Polyubiquitinated Cargo,” The EMBO Journal 29, no. 6 (2010): 1045–1054. 20150893 10.1038/emboj.2010.6 PMC2845278

[34] N. Gan , Y. Han , W. Zeng , et al., “TRPML1 Gating Modulation by Allosteric Mutations and Lipids,” Elife 13 (2024): RP100987. 39400550 10.7554/eLife.100987 PMC11473102

[35] B. Dong , X.‐Y. Liu , B. Li , M.‐Y. Li , S.‐G. Li , and S. Liu , “A Heat Shock Protein Protects Against Oxidative Stress Induced by Lambda‐cyhalothrin in the Green Peach Aphid Myzus persicae,” Pesticide Biochemistry and Physiology 181 (2022): 104995. 35082025 10.1016/j.pestbp.2021.104995

[36] T. Wu , Y. Sheng , Y. Tian , and C. Wang , “Vitexin Regulates Heat Shock Protein Expression by Modulating ROS Levels Thereby Protecting Against Heat‐Stress‐Induced Apoptosis,” Molecules (Basel, Switzerland) 28, no. 22 (2023): 7639. 38005362 10.3390/molecules28227639 PMC10675196

[37] A. Abdulsid and F. Lyall , “RETRACTED: Heat shock protein 27 expression is spatially distributed in human placenta and selectively regulated During preeclampsia,” Journal of Reproductive Immunology 101‐102 (2014): 89–95. 10.1016/j.jri.2013.09.002 24182452

[38] D. Singer , V. Ressel , M. B. Stope , and S. Bekeschus , “Heat Shock Protein 27 Affects Myeloid Cell Activation and Interaction With Prostate Cancer Cells,” Biomedicines 10, no. 9 (2022): 2192. 36140293 10.3390/biomedicines10092192 PMC9496253

[39] Y. Liang , Y. Wang , Y. Zhang , et al., “HSPB1 facilitates Chemoresistance Through Inhibiting Ferroptotic Cancer Cell Death and Regulating NF‐κB Signaling Pathway in Breast Cancer,” Cell Death & Disease 14, no. 7 (2023): 434. 37454220 10.1038/s41419-023-05972-0 PMC10349816

[40] S. Han , R. Wang , Y. Zhang , et al., “The Role of Ubiquitination and Deubiquitination in Tumor Invasion and Metastasis,” Review Int J Biol Sci. 18, no. 6 (2022): 2292–2303. 35414786 10.7150/ijbs.69411 PMC8990454

[41] K. Bokuda and A. Ichihara , “Preeclampsia up to Date—What's Going On?,” Hypertension Research 46, no. 8 (2023): 1900–1907. 37268721 10.1038/s41440-023-01323-w PMC10235860

[42] Q. Zhang , C.‐L. Lee , T. Yang , et al., “Adrenomedullin Has a Pivotal Role in Trophoblast Differentiation: A Promising Nanotechnology‐based Therapeutic Target for Early‐onset Preeclampsia,” Science Advances 9, no. 44 (2023): adi4777. 10.1126/sciadv.adi4777 PMC10624351 37922358

[43] M. Colombo , G. Raposo , and C. Thery , “Biogenesis, Secretion, and Intercellular Interactions of Exosomes and Other Extracellular Vesicles,” Annual Review of Cell and Developmental Biology 30 (2014): 255–289. 10.1146/annurev-cellbio-101512-122326 25288114

[44] F. Cichocki , B. Zhang , C.‐Y. Wu , et al., “Nicotinamide Enhances Natural Killer Cell Function and Yields Remissions in Patients With non‐Hodgkin Lymphoma,” Science Translational Medicine 15, no. 705 (2023): ade3341. 10.1126/scitranslmed.ade3341 PMC10859734 37467318

[45] F. Wang , A. E. Qualls , L. Marques‐Fernandez , and F. Colucci , “Biology and Pathology of the Uterine Microenvironment and Its Natural Killer Cells,” Cellular & Molecular Immunology 18, no. 9 (2021): 2101–2113. 34426671 10.1038/s41423-021-00739-z PMC8429689

[46] R. Wei , N. Lai , L. Zhao , et al., “Dendritic Cells in Pregnancy and Pregnancy‐Associated Diseases,” Biomedicine & Pharmacotherapy 133 (2021): 110921. 33378991 10.1016/j.biopha.2020.110921

[47] A.‐P. Cao , Y.‐Y. Wang , Y.‐Y. Shen , et al., “Nicotinamide Suppresses Hyperactivation of Dendritic Cells to Control Autoimmune Disease Through PARP Dependent Signaling,” Nutrients 16, no. 16 (2024): 2665. 39203802 10.3390/nu16162665 PMC11356829

[48] C. J. Nogueras‐Ortiz , E. Eren , P. Yao , et al., “Single‐Extracellular Vesicle (EV) Analyses Validate the Use of L1 Cell Adhesion Molecule (L1CAM) as a Reliable Biomarker of Neuron‐Derived EVs,” Journal of Extracellular Vesicles 13, no. 6 (2024): 12459. 10.1002/jev2.12459 PMC11170079 38868956

[49] N. Takahashi , F. Li , T. Fushima , et al., “Vitamin B_3_ Nicotinamide: A Promising Candidate for Treating Preeclampsia and Improving Fetal Growth,” The Tohoku Journal of Experimental Medicine 244, no. 3 (2018): 243–248. 29563389 10.1620/tjem.244.243 PMC7021450

[50] A. M. Real , S. Hong , and P. Pissios , “Nicotinamide N‐Oxidation by CYP2E1 in Human Liver Microsomes,” Drug Metabolism and Disposition 41, no. 3 (2013): 550–553. 23418369 10.1124/dmd.112.049734 PMC3583486

[51] B. Zhang , L. Tan , Y. Yu , et al., “Placenta‐Specific Drug Delivery by Trophoblast‐Targeted Nanoparticles in Mice,” Theranostics 8, no. 10 (2018): 2765–2781. 29774074 10.7150/thno.22904 PMC5957008

[52] Y. J. Lee , K. J. Shin , H.‐J. Jang , et al., “GPR143 Controls ESCRT‐Dependent Exosome Biogenesis and Promotes Cancer Metastasis,” Developmental Cell 58, no. 4 (2023): 320–334. 36800996 10.1016/j.devcel.2023.01.006

[53] W. Qin , C. Steinek , K. Kolobynina , et al., “Probing Protein Ubiquitination in Live Cells,” Nucleic Acids Research 50, no. 21 (2022): 125. 10.1093/nar/gkac805 PMC9757074 36189882

[54] H. SAHIN , T. GUNEL , A. BENIAN , E. ONAY UCAR , O. GURALP , and A. KILIC , “Genomic and Proteomic Investigation of Preeclampsia,” Experimental and Therapeutic Medicine 10, no. 2 (2015): 711–716. 26622380 10.3892/etm.2015.2509 PMC4509133

[55] T. Awoyemi , A. S. Cerdeira , W. Zhang , et al., “Preeclampsia and Syncytiotrophoblast Membrane Extracellular Vesicles (STB‐EVs),” Clinical Science (Lond) 136, no. 24 (2022): 1793–1807. 10.1042/CS20220149 PMC9751756 36511102

[56] J. H. Rowe , J. M. Ertelt , L. Xin , and S. S. Way , “Pregnancy Imprints Regulatory Memory That Sustains Anergy to Fetal Antigen,” Nature 490 (2012): 102–106. 23023128 10.1038/nature11462 PMC3465465

[57] J. Li , B. LaMarca , and J. F. Reckelhoff , “A Model of Preeclampsia in Rats: The Reduced Uterine Perfusion Pressure (RUPP) Model,” American Journal of Physiology‐Heart and Circulatory Physiology 303, no. 1 (2012): H1–H8. 22523250 10.1152/ajpheart.00117.2012 PMC3404644

[58] T. Lei , J. Zhang , Q. Zhang , et al., “Defining Newly Formed and Tissue‐Resident Bone Marrow‐Derived Macrophages in Adult Mice Based on Lysozyme Expression,” Cellular & Molecular Immunology 19, no. 12 (2022): 1333–1346. 36348079 10.1038/s41423-022-00936-4 PMC9708686

[59] X. Chen , F. Jia , Y. Li , et al., “Nitric Oxide‐induced Stromal Depletion for Improved Nanoparticle Penetration in Pancreatic Cancer Treatment,” Biomaterials 246 (2020): 119999. 32247201 10.1016/j.biomaterials.2020.119999

[60] X. Gao , Y. Fu , S. Sun , et al., “Cryptococcal Hsf3 Controls Intramitochondrial ROS Homeostasis by Regulating the respiratory Process,” Nature Communications 13, no. 1 (2022): 5407. 10.1038/s41467-022-33168-1 PMC9477856 36109512

