# Supplementary material for: HRS Degradation‐Induced Nicotinamide Deficiency in Placental Extracellular Vesicles Triggers Preeclampsia by Disrupting Maternal‐Fetal Immune Homeostasis
Source: Adv Sci (Weinh). 2026 Jan 14;13(11):e10188. doi: 10.1002/advs.202510188 (PMC12931225; doi:10.1002/advs.202510188)
Supplement: Supplementary file 1 — Supporting File: advs73263‐sup‐0001‐SuppMat.docx. [file ADVS-13-e10188-s001.docx]

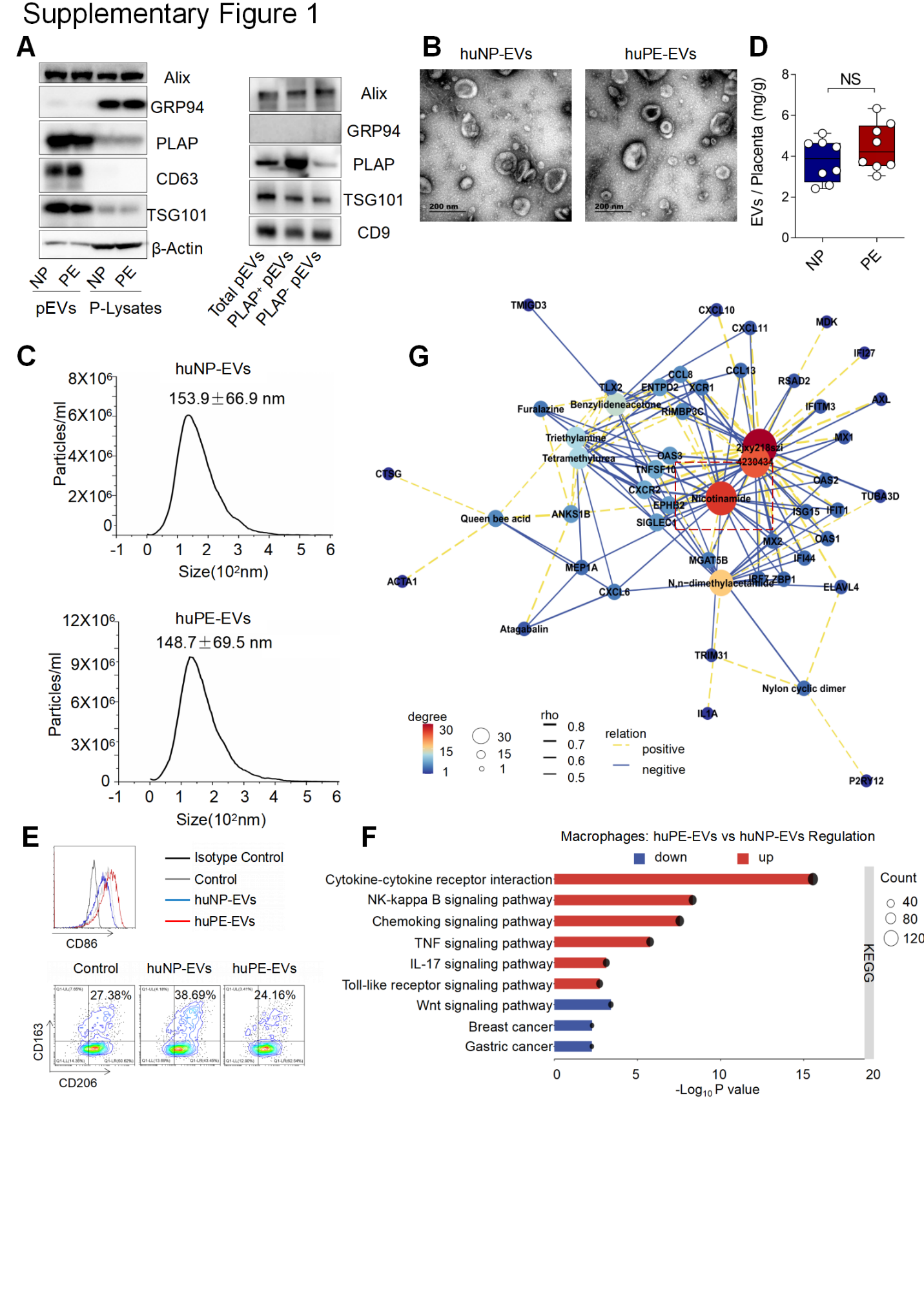


**Supplementary Figure1 Extraction and Identification of human pEVs**

(A) Placenta lysates and EVs proteins from women with NP and PE were analyzed by western blotting using indicated antibodies. (B) Representative TEM images of PALP^+^ EVs from NP and PE patients. Scale bar, 200 nm. (C) Concentration and size distribution of nano-sized particles in pEVs suspension were measured using nanoparticle tracking analysis. (D) Assessment of EVs Secretory Capacity in NP and PE patients’ placenta villus (n=8). (E) Frequencies of CD206^+^CD163^+^ cells and average fluorescence intensity of CD86 of macrophages treated with PBS (Control), NP-EVs or PE-EVs were analyzed by flow cytometry. (F) GO enrichment terms that were significantly enriched in the differentially expressed genes in macrophages treated with NP-EVs or PE-EVs. The red line indicates GO analysis of upregulated genes in the PE-EV group, while the blue line indicates GO analysis of downregulated genes in the PE-EV group. (G) A transcript-metabolic correlation network image of significantly altered metabolites in pEVs and selected significantly altered genes involved in the inflammatory response pathway in CD4^+^ T cells. The yellow dashed lines represent the positive correlation, while the blue solid lines represent the negative correlation between the metabolites and genes. Data normality was verified using Shapiro-Wilk test. The dataset in figure (D) exhibited non-normal distribution and was consequently analyzed using the Kruskal-Wallis test. All datas are presented as mean ± SEM (NS, not significant).


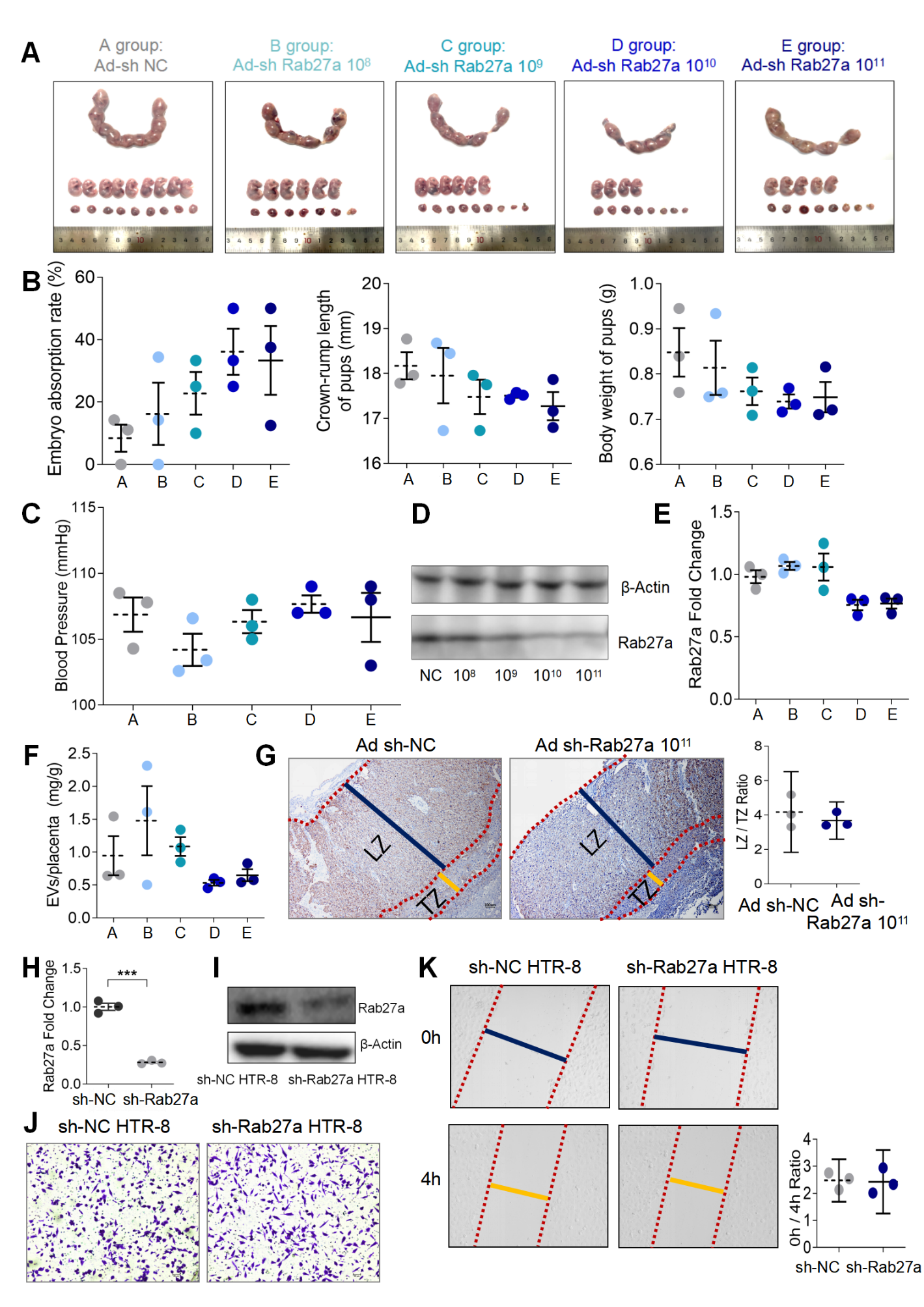


**Supplementary Figure2** **Animal model of intrauterine perfusion Rab27a adenovirus**

1. Mice were transfected with adenovirus-negative control (Ad-sh NC) and different concentration (10^8 pfu/ml, 10^9 pfu/ml, 10^10 pfu/ml and 10^11 pfu/ml) of Ad-Rab27a-shRNA (Ad-sh Rab27a). (B) Embryo abortion rate of the pregnant mice, body weight and crown-rump length of pups measured on day 18.5 of gestation. (C) SBP of pregnant mice in the five groups. (D) Detection of Rab27a Protein Expression in Mouse Placenta via Western Blot Analysis.(E) Detection of Rab27a RNA Expression in Mouse Placenta via qPCR Analysis. (F) Assessment of EVs Secretory Capacity in Mouse Placenta. (G) The placental labyrinth zone (LZ) to trophoblast zone (TZ) ratio in different mouse model group. (H) Detection of Rab27a RNA Expression in HTR-8 via qPCR Analysis. (I) Detection of Rab27a Protein Expression in HTR-8 via Western Blot Analysis. (J) Transwell-Matrigel assay of HTR8 cells treated with lentivirus-mediated si-Rab27A or NC. (K) Wound healing assay of HTR8 cells following lentivirus-mediated Rab27A knockdown or NC treatment. Data normality was verified using Shapiro-Wilk test. The dataset in this figure exhibited non-normal distribution and was consequently analyzed using the Kruskal-Wallis test.

**
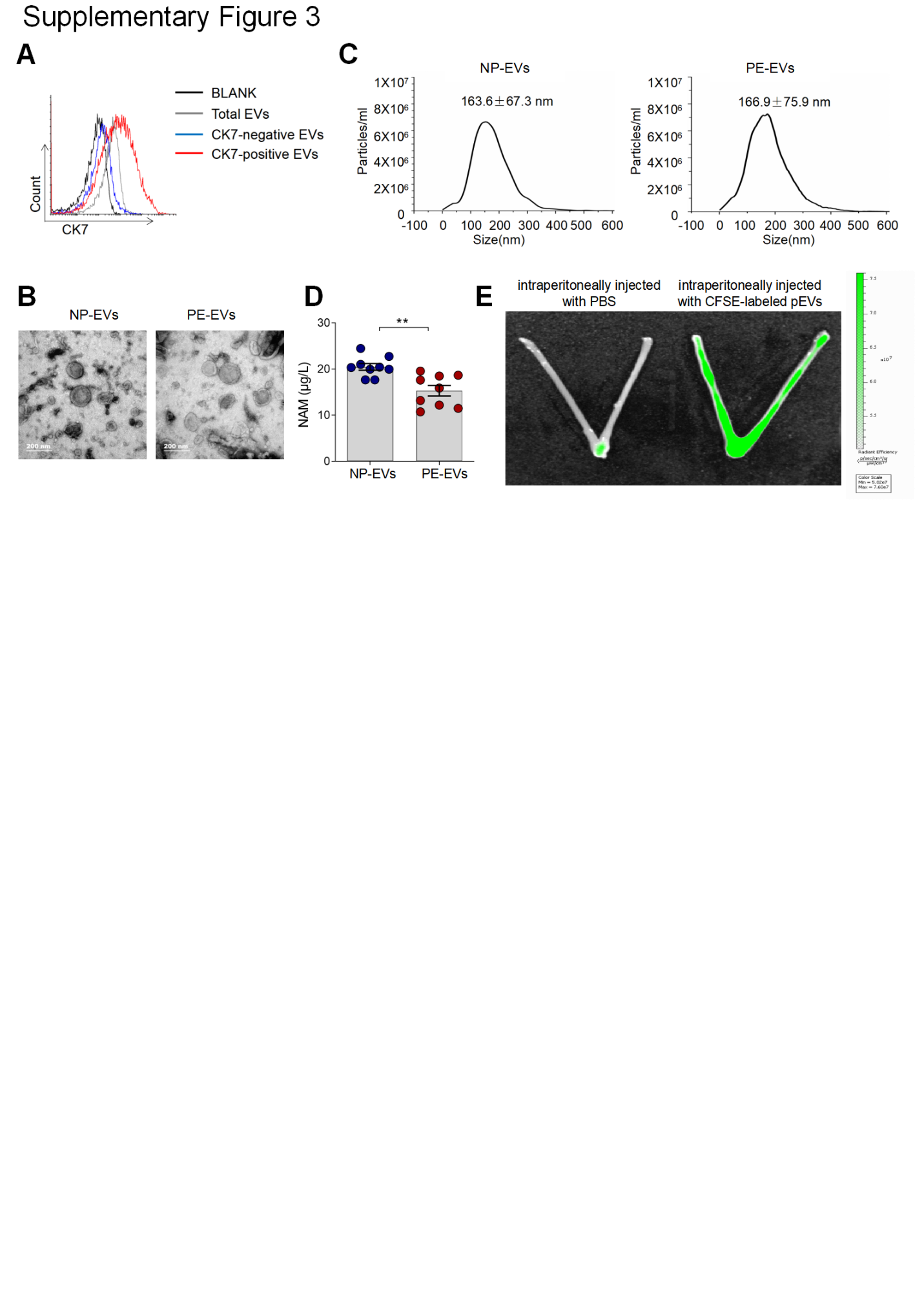
**

**Supplementary Figure3 Extraction and Identification of mouse pEVs.**

1. A representative FCM graph of the expression of CK7 in EVs. (B) Representative TEM images of CK7^+^ exosomes from mice with PE (PE-EVs) and those with NP (NP-EVs). Scale bar, 200 nm. (C) Concentration and size distribution of nano-sized particles in EVs suspension were measured using nanoparticle tracking analysis. (D) The concentration of NAM in mouse pEVs. (E) Following CFSE labeling, pEV distribution in the uterus was tracked after intraperitoneal injection using fluorescence imaging. Data normality was verified using Shapiro-Wilk test. The dataset in this figure exhibited normal distribution and were compared between groups using unpaired t-test and represented as mean±SEM (** P < 0.01).

**
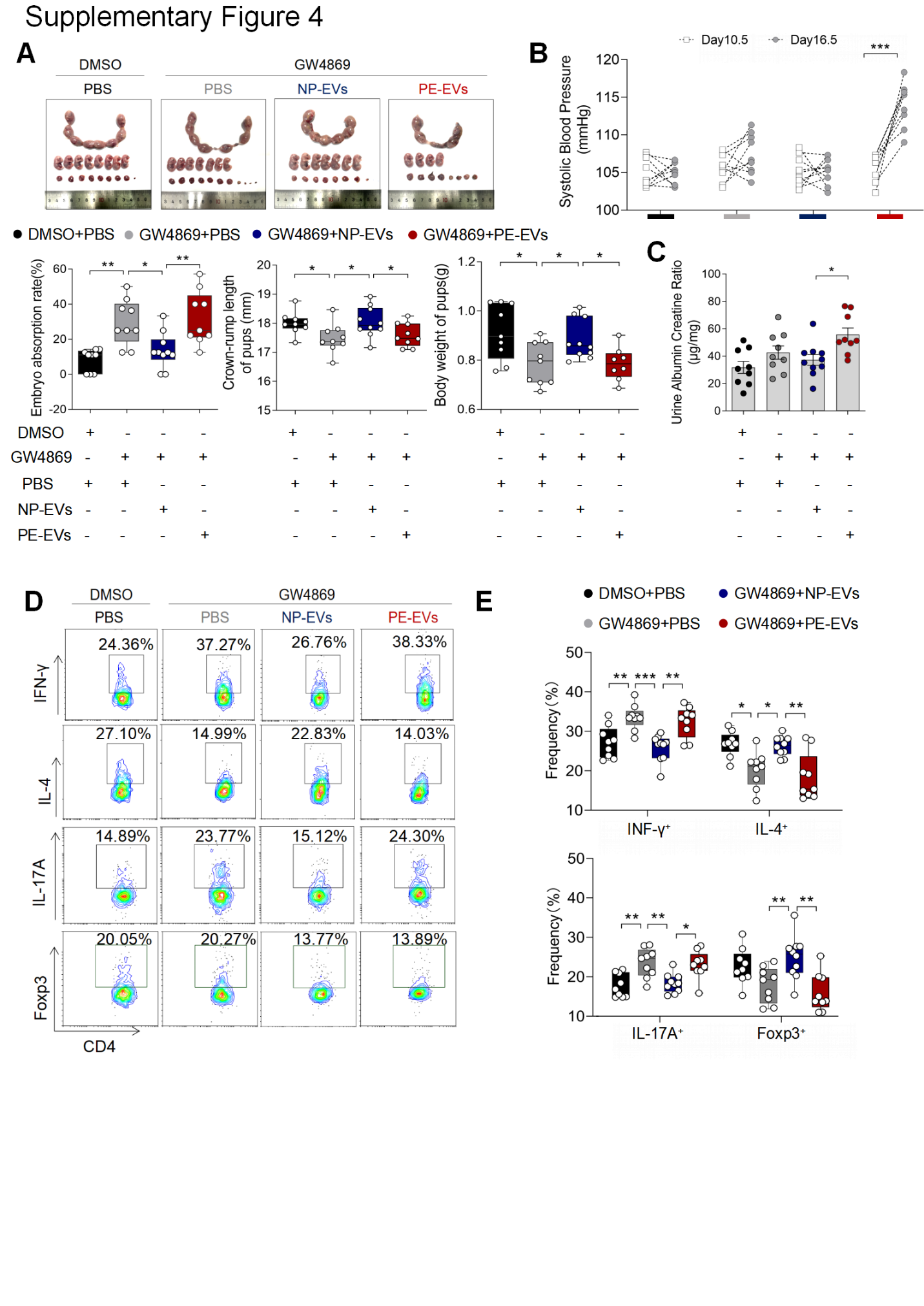
**

**Supplementary Figure4 Animal model of intrauterine perfusion GW4869**

1. Embryo abortion rate of the pregnant mice, body weight and crown-rump length of pups measured on day 18.5 of gestation. (B) SBP of pregnant mice in the four groups . (C) UACR in urine of pregnant mice in the four groups. (D) Frequencies of IFN-γ^+^CD4^+^, IL-4^+^CD4^+^, IL-17A^+^CD4^+^, Foxp3^+^CD4^+^ T cells were analyzed by flow cytometry. (E) Statistical analysis of frequencies of IFN-γ^+^CD4^+^, IL-4^+^CD4^+^, IL-17A^+^CD4^+^, Foxp3^+^CD4^+^ T cells. Data normality was verified using Shapiro-Wilk test. The dataset in figure (B) exhibited non-normal distribution and was consequently analyzed using the Kruskal-Wallis test, the normally distributed data in other figures were evaluated by one-way ANOVA. All datas are presented as mean ± SEM (*P < 0.05, ** P < 0.01, *** P < 0.001).

**
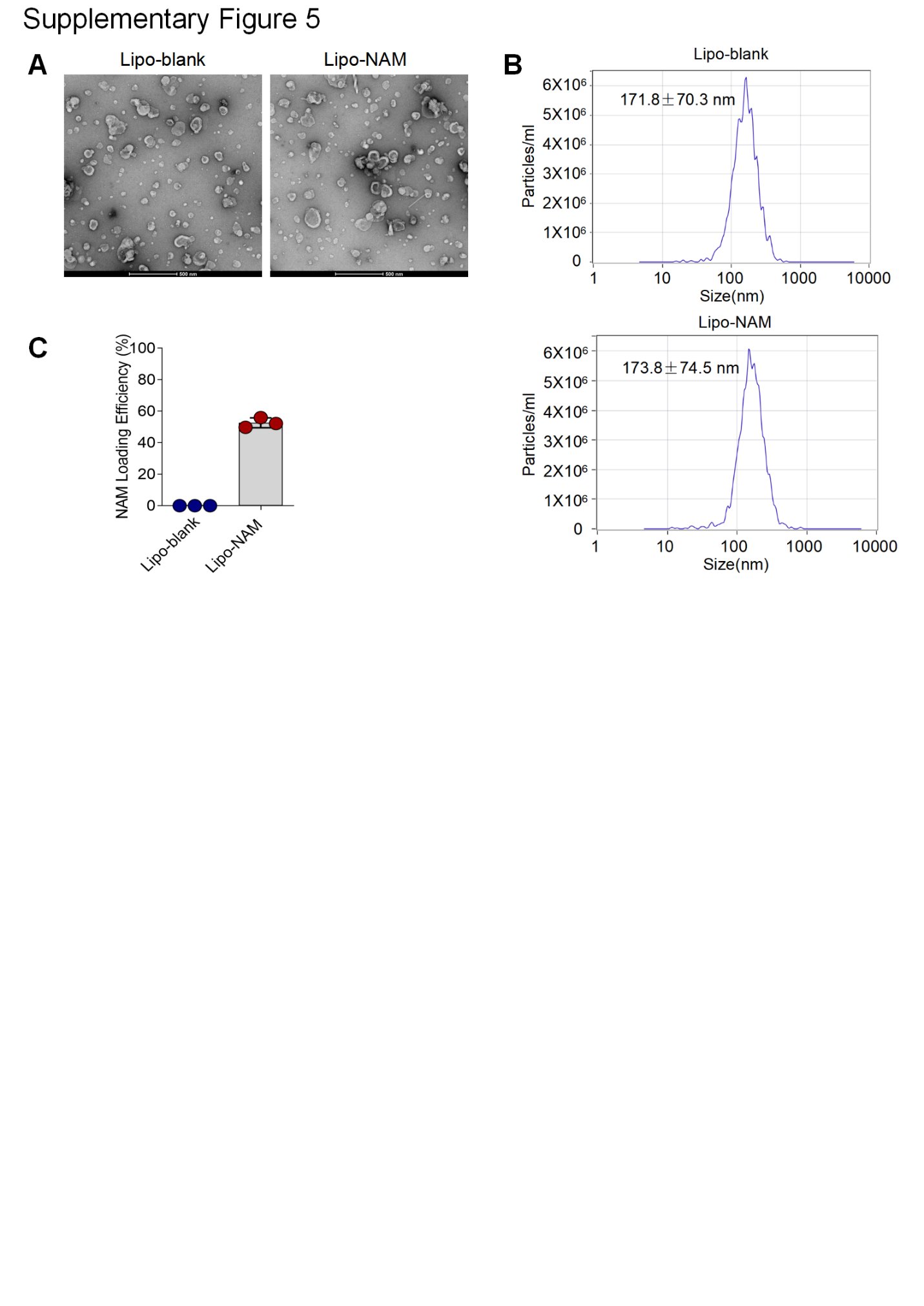
Supplementary Figure5 Identification of Lipo-NAM**

1. Representative TEM images of Lipo-blank and Lipo-NAM. Scale bar, 500 nm. (B) Concentration and size distribution of nano-sized particles in Lipo-blank and Lipo-NAM suspension were measured using nanoparticle tracking analysis. (C) The NAM loading efficiency of liposomes was calculated using the following equation: The NAM loading efficiency (%) = [Amount of NAM in liposomes] / [Amount of NAM in liposomes + Amount of NAM in supernatant] × 100.


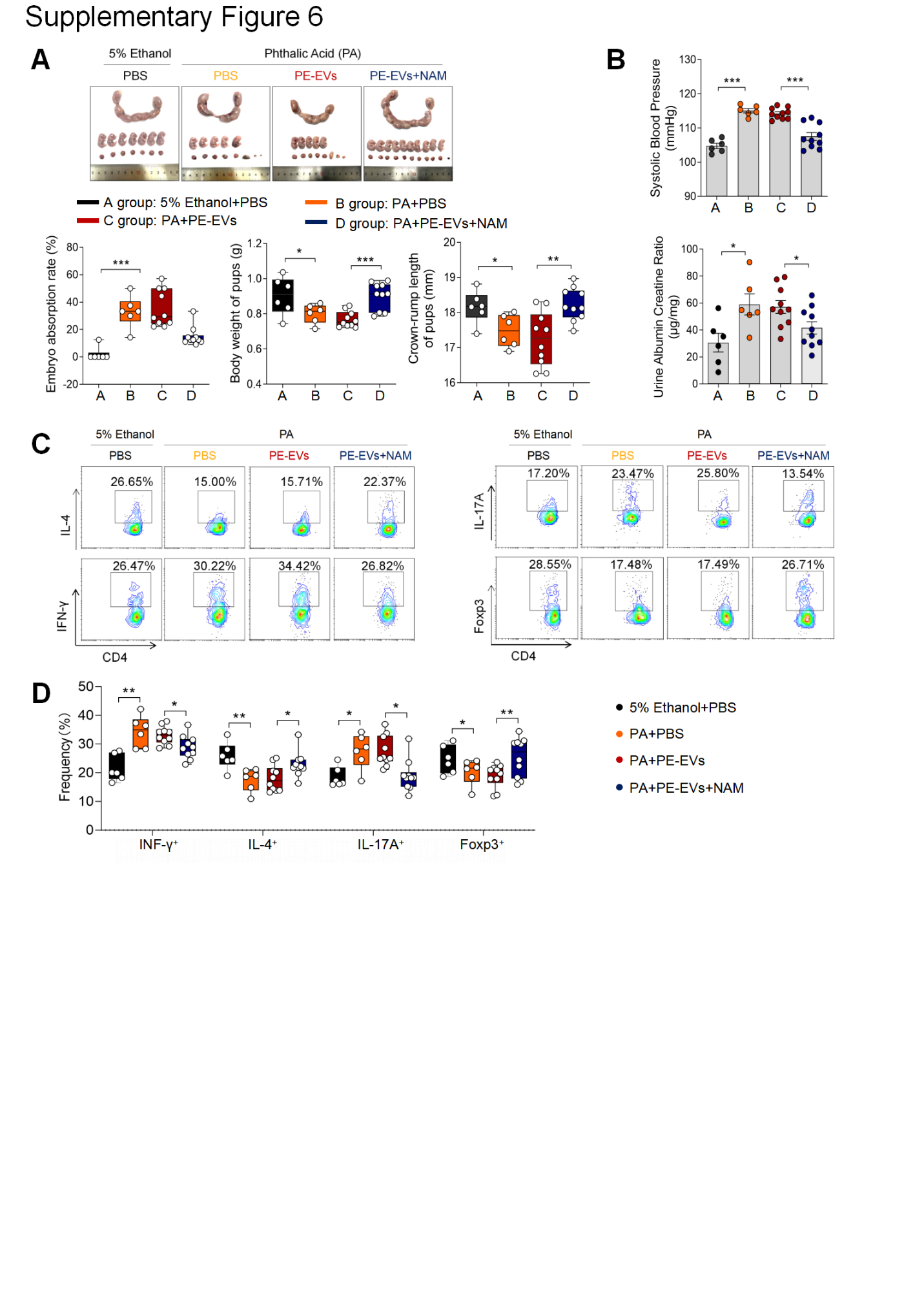


**Supplementary Figure6 Administration of NAM Enhances Pregnancy Outcomes in PA-induced PE Mouse Models**

Embryo abortion rate of the pregnant mice, body weight and crown-rump length of pups measured on day 18.5 of gestation. Black represents mice intrauterine infusion of 5% Ethanol accompanied by intraperitoneal injection of PBS (the number of pregnant mice: 6; the number of fetuses per pups of each pregnant mice: 7, 7, 9, 7, 8, 8); Orange represents mice intrauterine infusion of PA accompanied by intraperitoneal injection of PBS (the number of pregnant mice: 6; the number of fetuses per pups of each pregnant mice: 5, 5, 7, 6, 2, 3); Red represents mice intrauterine infusion of PA accompanied by intraperitoneal injection of PE-EVs (the number of pregnant mice: 10; the number of fetuses per pups of each pregnant mice: 7, 3, 1, 3, 7, 6, 6, 6, 5, 7); blue represents mice intrauterine infusion of PA accompanied by intraperitoneal injection of PE-EVs and NAM (the number of pregnant mice: 10; the number of fetuses per pups of each pregnant mice: 8, 7, 8, 6, 6, 4, 7,10, 6, 7). (B) SBP and UACR of the pregnant mice. (C)Frequencies of IFN-γ^+^CD4^+^, IL-4^+^CD4^+^, IL-17A^+^CD4^+^, Foxp3^+^CD4^+^ T cells were analyzed by flow cytometry. (D) Statistical analysis of the frequencies of Th1, Th2, Th17 and Treg cells. Data normality was verified using Shapiro-Wilk test. The dataset in figure (B)’ UACR, figure (D)’ IFN-γ and IL-4 exhibited non-normal distribution and was consequently analyzed using the Kruskal-Wallis test. The normally distributed data in other figures were evaluated by Student's t-test (two groups) or one-way ANOVA (multiple groups). All datas are presented as mean ± SEM (*P < 0.05, ** P < 0.01, *** P < 0.001).


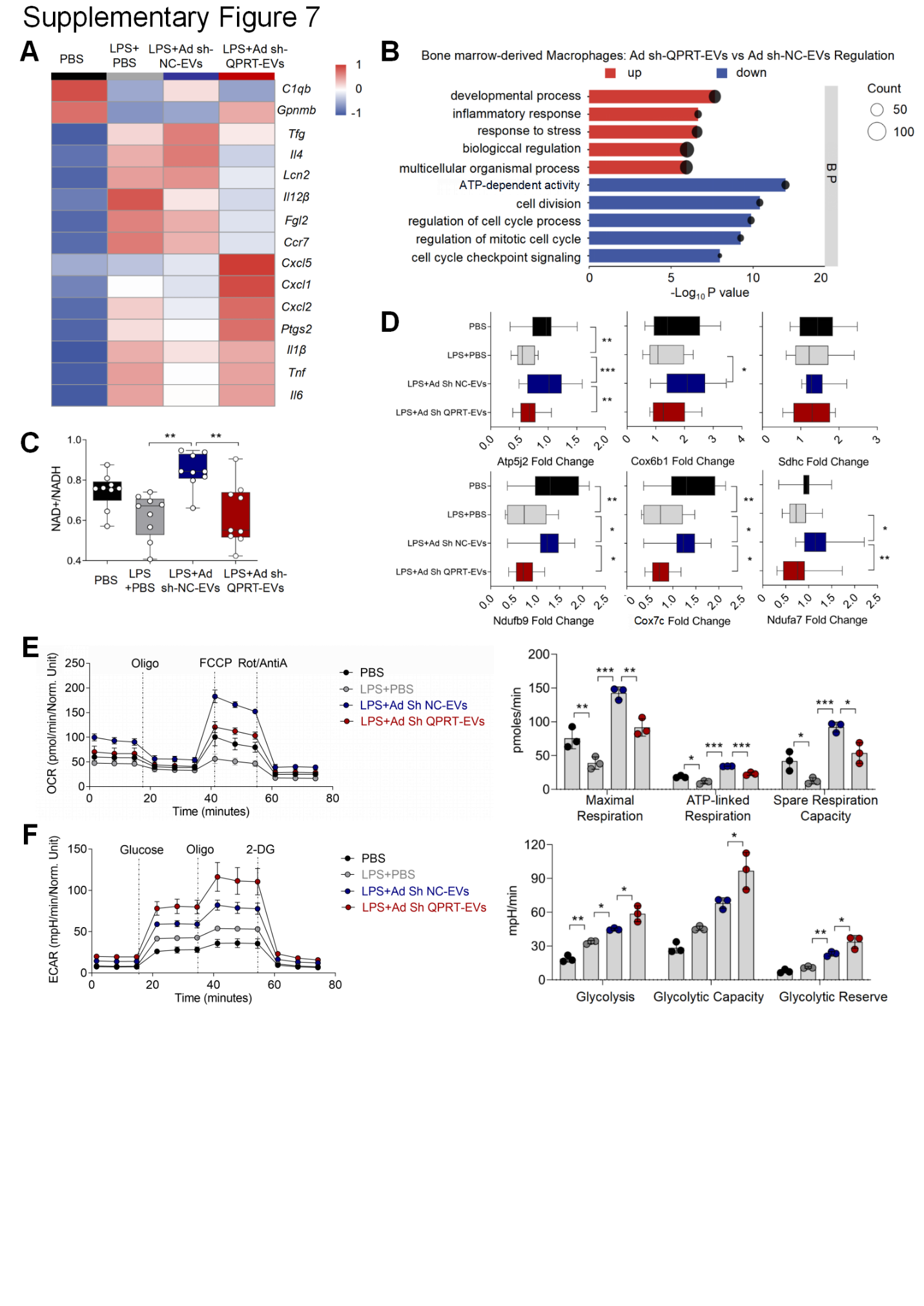


**Supplementary Figure7 pEVs enhances oxidative phosphorylation via NAM, thereby inhibiting the polarization of pro-inflammatory macrophages**

1. The heatmap shows differential gene expression of BMDMs treated with Ad Sh-NC-EVs or Ad Sh-*QPRT*-EVs. (B) GO enrichment terms that were significantly enriched in the differentially expressed genes in BMDMs treated with Ad Sh-NC-EVs or Ad Sh-*QPRT*-EVs. The red line indicates GO analysis of upregulated genes in the Ad Sh-*QPRT*-EVs group, while the blue line indicates GO analysis of downregulated genes in the Ad Sh-*QPRT*-EVs group. (C) NAD+/NADH levels in BMDMs. (D) Changes of the oxidative phosphorylation-related gene expression in BMDMs. (E) The OCR profiles (the average maximal respiration, ATP-linked respiration, and SRC) of BMDMs treated with Ad Sh-NC-EVs or Ad Sh-*QPRT*-EVs. (F) The ECAR profiles (Glycolysis, Glycolytic Capacity and Glycolytic Reserve) of BMDMs treated with Ad Sh-NC-EVs or Ad Sh-*QPRT*-EVs. Data normality was verified using Shapiro-Wilk test. The dataset in figure (E) and (F) exhibited non-normal distribution and was consequently analyzed using the Kruskal-Wallis test. The normally distributed data in other figures were evaluated by one-way ANOVA. All datas are presented as mean ± SEM (*P < 0.05, ** P < 0.01, *** P < 0.001).

**
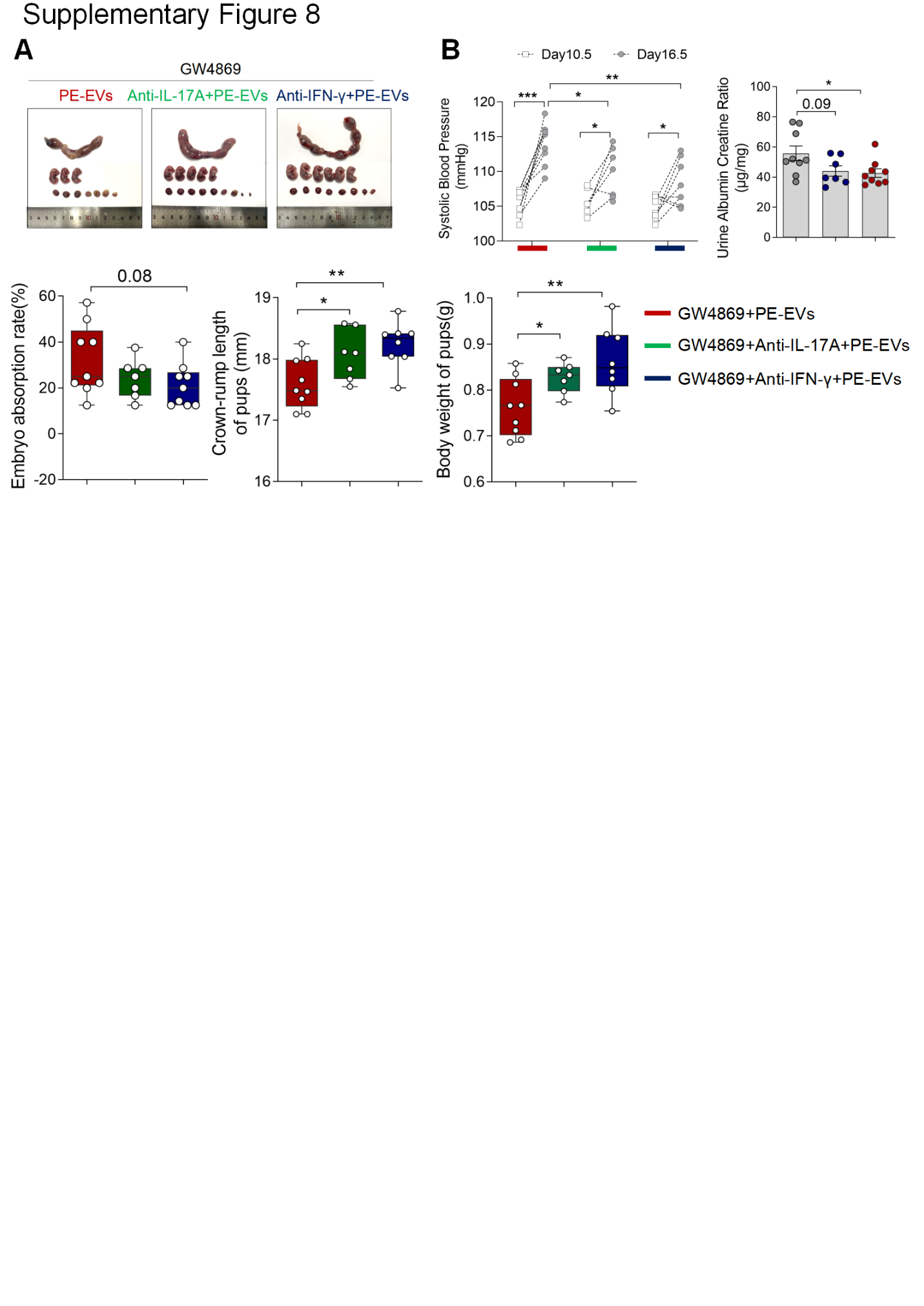
**

**Supplementary Figure8 Th1 Cells Dominance Mediates pEV-Induced PE Pathogenesis**

1. Embryo abortion rate of the pregnant mice, body weight and crown-rump length of pups measured on day 18.5 of gestation. Red represents mice intrauterine infusion of GW4869 accompanied by intraperitoneal injection of PE-EVs (the number of pregnant mice: 9; the number of fetuses per pups of each pregnant mice: 4, 7, 8, 3, 6, 7, 7, 6, 6); Green represents mice intrauterine infusion of GW4869 accompanied by intraperitoneal injection of PE-EVs after IL-17A neutralization (the number of pregnant mice: 7; the number of fetuses per pups of each pregnant mice: 5, 4, 5, 6, 5, 5, 5); Blue represents mice intrauterine infusion of GW4869 accompanied by intraperitoneal injection of PE-EVs after INF-γ neutralization (the number of pregnant mice: 8; the number of fetuses per pups of each pregnant mice: 6, 7, 7, 7, 6, 6, 7, 8 ). (B) SBP and UACR of pregnant mice in the three groups. Data normality was verified using Shapiro-Wilk test. The dataset in figure (B)’ UACR exhibited non-normal distribution and was consequently analyzed using the Kruskal-Wallis test. The normally distributed data in other figures were evaluated by one-way ANOVA . All datas are presented as mean ± SEM (*P < 0.05).


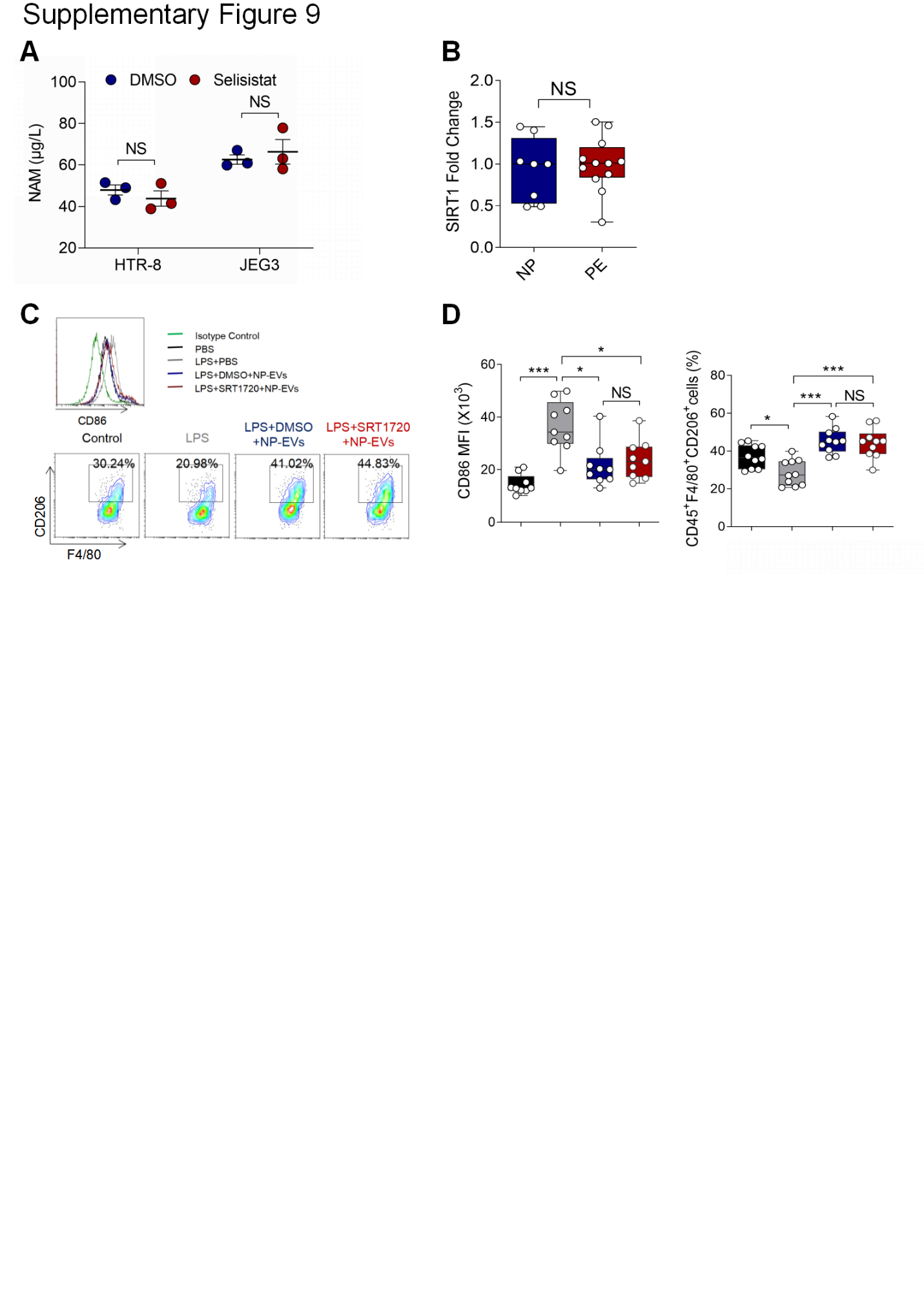


**Supplementary Figure9 SIRT1 Activation by Selisistat Fails to Modulate NAM Levels in pEVs**

1. NAM levels in HTR-8 and JEG3 treated with DMSO or selisistat. (B) SIRT1 mRNA expression levels in placentas from NP and PE patients. (C) Frequencies of CD206^+^F4/80^+^ cells and average fluorescence intensity of CD86 were analyzed by flow cytometry. (D) Statistical analysis of the frequencies of CD206^+^F4/80^+^ cells and average fluorescence intensity of CD86.

**
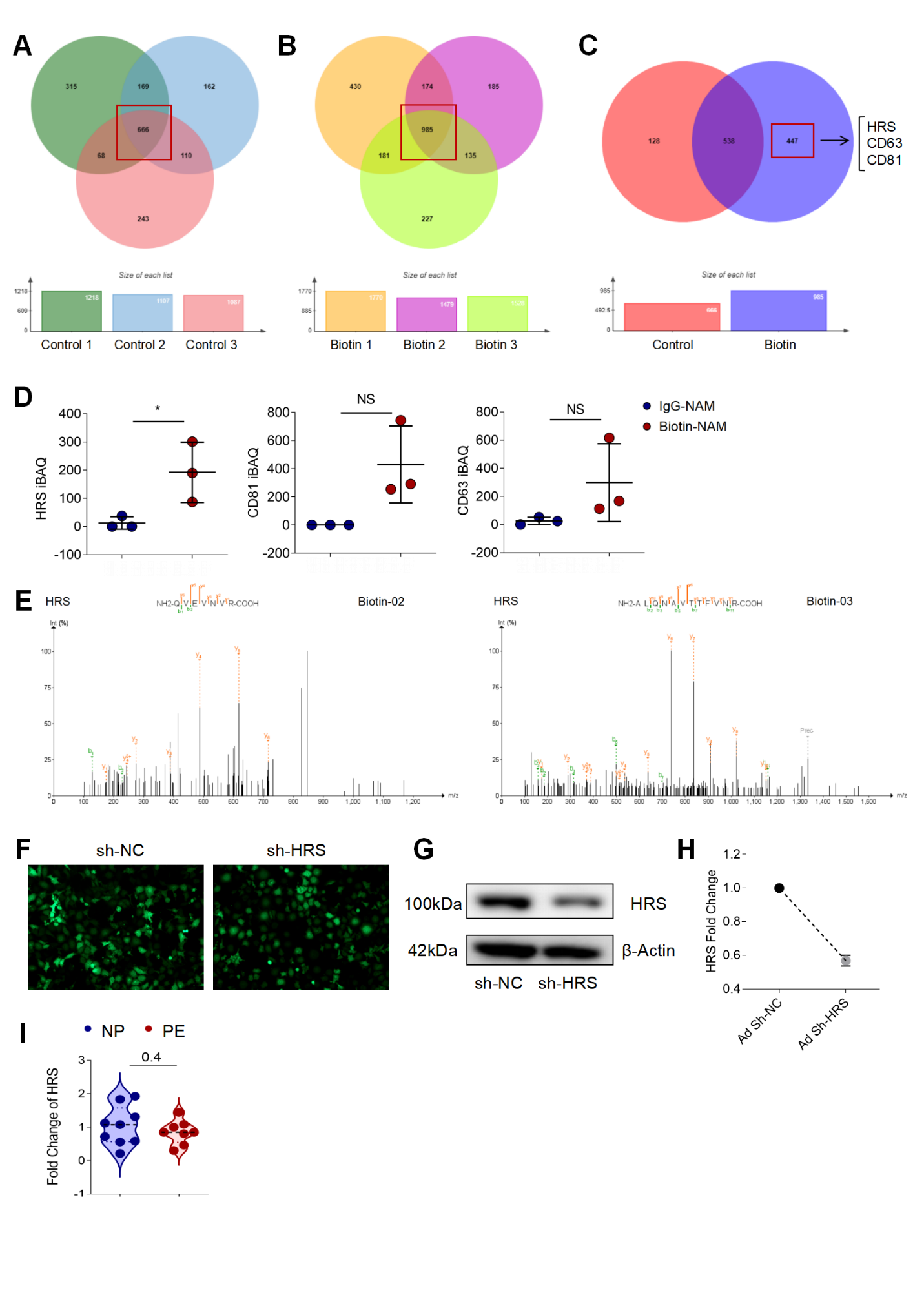
**

**Supplementary Figure10 Detection of NAM-bound proteins by mass spectrometry**

(A) Numbers of proteins detected in the Control group were shown by Venn diagram. Proteins expressed in all samples were highlighted. (B) Numbers of proteins detected in the Biotin group were shown by Venn diagram. Proteins expressed in all samples were highlighted. (C) Proteins highlighted in A and B were contrasted and those especially expressed in the Biotin group were highlighted which contain HRS, CD63 and CD81. (D) The iBAQ of HRS, CD63 and CD81 between Biotin and Control group were statistically analyzed. (E) Mass spectrometry analysis of the different proteins in other placenta explants treated with con-NAM and NAM-biotin. (F) Fluorescence intensity of trophoblast cells after lentiviral transfection detected by fluorescence microscopy. (G) Detection of HRS protein Expression in Mouse Placenta via Western blot Analysis. (H) Detection of HRS RNA Expression in Mouse Placenta via qPCR Analysis. (I) RT-PCR analysis of HRS mRNA levels in placental villus explant from NP and PE patients.


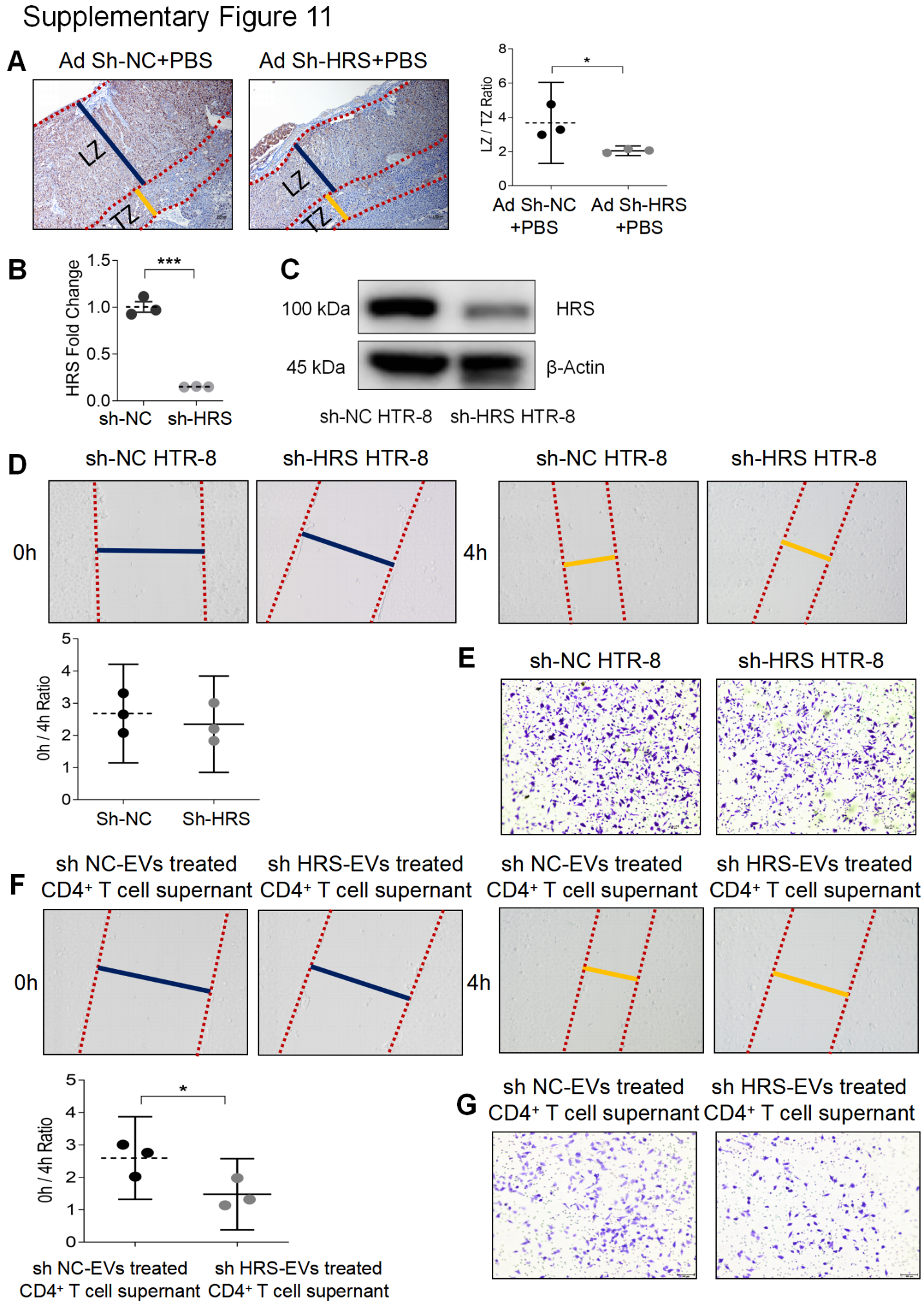


**Supplementary Figure11 HRS deficiency impairs trophoblast invasion in vivo indirectly**

1. The placental labyrinth zone (LZ) to trophoblast zone (TZ) ratio in different mouse model group. (B) Detection of HRS RNA Expression in HTR-8 via qPCR Analysis. (C) Detection of HRS Protein Expression in HTR-8 via Western Blot Analysis. (D) Wound healing assays showing the migratory capacity of HTR8 cells after lentivirus-mediated HRS knockdown or NC treatment.(E) Transwell-Matrigel assays showing HTR8 cell migration after treatment with lentivirus-mediated si-HRS or NC. (F) Wound healing assay showing HTR8 cell migration after coculture with supernatant from CD4^+^ T cells that were treated with sh-NC-EVs or sh-HRS-EVs. (G) Transwell-Matrigel assays showing HTR8 cell migration after coculture with supernatant from CD4^+^ T cells pre-treated with sh-NC-EVs or sh-HRS-EVs.
